# Evaluating GPT-4’s role in critical patient management in emergency departments

**DOI:** 10.1371/journal.pone.0327584

**Published:** 2025-07-24

**Authors:** Yavuz Yiğit, Serkan Günay, Ahmet Öztürk, Baha Alkahlout

**Affiliations:** 1 Blizard Institute, Queen Mary University, London, United Kingdom; 2 Hamad Medical Corporation, Department of Emergency Medicine, Doha, Qatar; 3 Hitit University, Çorum Erol Olçok Education and Research Hospital, Department of Emergency Medicine, Çorum, Turkey; Dokkyo Medical University, JAPAN

## Abstract

**Introduction:**

Recent advancements in artificial intelligence (AI) have introduced tools like ChatGPT-4, capable of interpreting visual data, including ECGs. In our study,we aimed to investigate the effectiveness of GPT-4 in interpreting ECGs and managing patient care in emergency settings.

**Methods:**

Conducted from April to May 2024, this study evaluated GPT-4 using twenty case scenarios sourced from PubMed Central and the OSCE sample question book. These cases, categorized into common and rare scenarios, were analyzed by GPT-4, and its interpretations were reviewed by five experienced emergency medicine specialists. The accuracy of ECG interpretations and subsequent patient management plans were assessed using a structured evaluation framework and critical error identification.

**Results:**

GPT-4 made critical errors in 46% of ECG interpretations in the OSCE group and 50% in the PubMed group. For patient management, critical errors were found in 32% of the OSCE group and 14% of the PubMed group. When ECG evaluations were included in patient management, error rates approached 50%. The inter-rater reliability among evaluators indicated good agreement (ICC = 0.725, F = 3.72, p < 0.001).

**Conclusion:**

While GPT-4 shows promise in specific applications, its current limitations in accurately interpreting ECGs and managing critical patient scenarios render it inappropriate for emergency department use. Future improvements and extensive validations are essential before such AI tools can be reliably deployed in critical healthcare settings.

## Introduction

Emergency departments play a pivotal role in the management of critically ill patients, where the use of electrocardiography (ECG) is essential [1]. With the recent advancements in technology, artificial intelligence (AI)-assisted ECG evaluation tools have begun to emerge [2]. In recent years, the integration of chatbots powered by large language models (LLMs) into our daily lives has gained significant traction. The potential of these LLMs to accurately interpret ECGs presents an intriguing area of study [3].

Chat-GPT, developed by OpenAI, is one of the leading Large Language Models (LLMs) available for user interaction. One of the latest versions, GPT-4, has the ability to process and interpret visual data [4]. Several studies have explored the application of GPT-4 in contemporary medical practice, including areas such as ECG evaluation and critical patient management [5–9]. One study assessed the accuracy of GPT-4 in ECG interpretation, finding that it correctly answered approximately two-thirds of the questions posed [5]. Another study noted that GPT-4’s performance diminished in non-multiple-choice questions [6]. Furthermore, research on GPT-4’s effectiveness in managing cardiac arrest and bradycardia patients highlighted that some critical steps were occasionally omitted [9]. The literature on the use of GPT-4 in patient management and ECG evaluation in emergency departments is still limited. Investigating the usability of GPT-4 for patient management and ECG evaluation in emergency department contexts could significantly benefit physicians operating under demanding conditions.

The primary aim of our research is to evaluate the efficacy of GPT-4 in the assessment of electrocardiograms (ECGs) within emergency department settings and to determine its effectiveness in patient management based on these evaluations.Additionally, the secondary aim is to compare the effectiveness of GPT-4 between more commonly encountered cases and rarer or more challenging cases.

## Materials and methods

This study was conducted between April 1, 2024, and May 31, 2024. As no patient data was utilized, ethical approval was not required. Our study was conducted in three phases: the first phase involved submitting clinical cases and ECGs to GPT-4; the second consisted of expert evaluations of GPT-4’s ECG interpretations and patient management suggestions; and the third phase focused on identifying critical errors in GPT-4’s responses. Twenty case examples were identified from two sources: PubMed Central and the Objective Structured Clinical Examinations (OSCE) sample question book, with ten cases from each source. PubMed Central provided rare or more challenging cases, while OSCE questions are designed to assess students’ clinical knowledge and consist of moderate, more commonly encountered scenarios [10]. Ten cases were selected from the PubMed database by searching for “resuscitation,” “ECG,” and “emergency medicine” between April 1, 2024, and April 7, 2024 [11–20]. Cases were screened for inclusion if they contained an ECG image and a clinical scenario reflecting emergency presentations (e.g., cardiac arrest, arrhythmia, STEMI). Ten cases were selected based on clarity, quality of ECG image, and clinical relevance. For the OSCE group, ten cases were selected from a widely used clinical question book, prioritizing common emergency ECG scenarios [21]. Two independent reviewers selected the cases, with final inclusion determined through consensus.

Given the exploratory nature of this study and the lack of prior validated performance data for ChatGPT-4 in visual ECG interpretation, a formal sample size calculation was not performed. Instead, a pragmatic sample of 20 cases was selected to ensure representation of both common and complex emergency scenarios and to allow comparative subgroup analysis. This number also provided sufficient data points (20 cases × 5 evaluators × 2 domains) to conduct meaningful statistical analysis.

These sample cases were evaluated by GPT-4 between April 11, 2024, and April 20, 2024. The version of GPT-4 capable of evaluating visual data dated November 6, 2023, was used for the evaluations. Separate chat pages were used for each evaluation. Prior to the evaluation, GPT-4 was instructed with the command, “You are an emergency medicine specialist working in an emergency department. Evaluate the following ECG and case accordingly.” Following this, the ECG image was uploaded, and the command “Describe this ECG” was issued, with the responses recorded. Subsequently, the clinical scenario was provided along with the instruction, “The patient presenting with/being brought in with the above-described ECG has the following clinical scenario. Manage the patient appropriately,” and the responses were recorded. The cases and the responses provided by GPT-4 are detailed in the Appendix.

In the second phase of the study, five emergency medicine specialists, each with a minimum of five years of experience, evaluated the ECG interpretations and patient management plans provided by GPT-4. This evaluation took place between April 20, 2024, and April 30, 2024, and was conducted in three categories:

1. The correctness of the ECG interpretations was assessed and categorized as follows: Correct (C) for accurate ECG interpretation, Largely Correct (LC) for largely accurate interpretation with some errors or omissions, Largely Incorrect (LIC) for partially correct interpretation but largely erroneous or omitted, and Incorrect (IC) for incorrect ECG interpretation.
2. Assuming the ECG interpretation was correct, the patient management (Without ECG group) was evaluated using a 7-point Likert scale (1: critical errors, substandard care; 2: numerous errors, inadequate care; 3: notable errors, insufficient care; 4: some errors, acceptable care; 5: minor errors, effective care; 6: slight errors, highly effective care; 7: no errors, exemplary care).
3. Taking into account the correctness or incorrectness of the ECG interpretation, the patient management (Within ECG group) was evaluated using the same 7-point Likert scale (1: critical errors, substandard care; 2: numerous errors, inadequate care; 3: notable errors, insufficient care; 4: some errors, acceptable care; 5: minor errors, effective care; 6: slight errors, highly effective care; 7: no errors, exemplary care). Evaluators were aware of the final diagnoses for each case during the assessment process. Providing the final diagnosis to evaluators ensured that their assessments were guided by the correct clinical context, reducing the risk of misclassification due to ambiguity or personal interpretation variability.

In the third phase of the study, evaluators were asked to assess whether there were any critical errors in both the ECG interpretations and case managements for each case. A critical error was defined as any mistake in case management or ECG interpretation that could potentially lead to morbidity or mortality.

GPT-4 responses were sent to evaluators randomly through Google Documents, ensuring blinding regarding which cases were from the OSCE book and which were from PubMed. Evaluators were also unaware of each other’s responses. Written consent was obtained from each evaluator before the evaluation. The responses were recorded and statistically analyzed.

The selection of five expert evaluators was based on balancing feasibility with sufficient inter-rater diversity. Inter-rater reliability was assessed using the Intraclass Correlation Coefficient (ICC), which demonstrated good agreement among evaluators.

Based on preliminary observations and informal testing, we expected GPT-4 to demonstrate a critical error rate of approximately 20–30% in complex emergency scenarios. This assumption guided the structure of our evaluation process and has been stated in the revised manuscript.

### Statistical analysis

Statistical analyses were performed using IBM SPSS software, version 25. The normality of variable distributions was examined using the Shapiro-Wilk test. Descriptive analyses were presented as mean and standard deviation for normally distributed variables and median and interquartile ranges for non-normally distributed variables. The Student-T test was used for normally distributed variables, and the Mann-Whitney U test was used for non-normally distributed variables. The Chi-square test was used for nominal variables. The inter-rater reliability was assessed using the Intraclass Correlation Coefficient (ICC) test. A p-value of less than 0.05 was considered statistically significant.

## Results

In our study, statistically significant differences were observed in the management evaluations for cases 1 (p = 0.05), 7 (p = 0.039), and 8 (p = 0.030) in the OSCE group, both with and without ECG evaluation. No significant differences were found in the management of other cases. In the PubMed group, statistically significant differences were found in the management of cases 7 (p = 0.025) and 9 (p = 0.025), while no significant differences were found in the management of other cases. Detailed information is shown in Table 1. When responses to all cases were evaluated collectively, it was observed that GPT-4’s case management was statistically more successful in the group without ECG evaluation compared to the group with ECG evaluation in the PubMed case group (p < 0.001). Similarly, in the OSCE group, it was found that the without ECG group was statistically more successful compared to the within ECG group (p < 0.001). Detailed information is shown in Table 2.

**Table 1:** Evaluation of OSCE and PubMed cases according to Within ECG and Without ECG groups.

Group	Cases	Within ECG	Without ECG	%95 CI	P value
OSCE	C1_mean±sd_	4.20 ± 1.01	5.80 ± 1.01	−3.20, −0.002	**0.050** ^*^
	C2_median, IQR (%25–75)_	7, 1(6–7)	7, 1(6–7)	−2.00, 2.00	1.000^**^
	C3_mean±sd_	4.80 ± 1.64	5.60 ± 1.14	−2.86, 1.31	0.894^*^
	C4_median, IQR (%25–75)_	6, 2(45–6)	6, 0(6–6)	−3.00, 1.00	0.408^**^
	C5_mean±sd_	4.80 ± 1.30	6.00 ± 0.00	−3.01, 1.01	0.109^***^
	C6_mean±sd_	6.00 ± 1.23	6.00 ± 1.23	−1.79, 1.79	1.000^*^
	C7_mean±sd_	2.60 ± 1.14	4.80 ± 1.64	−4.26, −0.14	**0.039** ^*^
	C8_median, IQR (%25–75)_	2, 0(2–2)	6, 4 (3 –7 )	−5.00, −1.00	**0.030** ^**^
	C9_mean±sd_	3.00 ± 1.23	4.60 ± 2.41	−4.56, 1.19	0.234^***^
	C10_mean±sd_	3.60 ± 1.82	5.54 ± 1.34	−4.13, 0.53	0.113^*^
PubMed	C1_median, IQR (%25–75)_	3, 0 (3 –3 )	6, 2 (4 –6 )	−4.00, 1.00	0.163^**^
	C2_median, IQR (%25–75)_	5, 1(5–6)	6, 0(6–6)	−3.00, 0.00	0.059^**^
	C3_median, IQR (%25–75)_	5, 1(5–6)	6, 0(6–6)	−3.00, 1.00	0.217^**^
	C4_mean±sd_	4.40 ± 1.82	6.00 ± 0.00	−3.86, 0.66	0.120^***^
	C5_mean±sd_	3.40 ± 2.07	4.60 ± 2.41	−4.48, 2.08	0.423^*^
	C6_median, IQR (%25–75)_	6, 0(6–6)	6, 0(6–6)	−1.00, 1.00	1.000^**^
	C7_median, IQR (%25–75)_	4, 1(3–4)	6, 0(6–6)	−4.00, −2.00	**0.025** ^**^
	C8_median, IQR (%25–75)_	5, 0(5–5)	6, 1(5–6)	−2.00, 1.00	0.434^**^
	C9_median, IQR (%25–75)_	5, 2 (3 –5 )	6, 0(6–6)	−5.00, −1.00	**0.025** ^**^
	C10_mean±sd_	5.60 ± 1.52	6.20 ± 0.84	−3.00, 1.00	0.461^*^

Within ECG: Case management with ECG response, Without ECG: The ECG response given is considered correct and only case management, OSCE: cases obtained from objective structured clinical examination (OSCE) questions, PubMed: Cases obtained from case reports in PubMed C:case, IQR: Inter quartile ratio, CI: Confidence interval.

p < 0.05 was considered statistically significant.

* According to Student T test results.

** According to Mann-Whitney U test results.

*** According to Welch test results.

**Table 2:** Evaluation of all OSCE and PubMed cases according to Within ECG and Without ECG groups.

	OSCE			PubMed		
	Median	IQR (%25, %75)	P Value^*^	Median	IQR (%25, %75)	P Value^*^
Within ECG	4.00	3.00 (3.00,6.00)	**<0.001**	5.00	3.00 (3.00-6.00)	**<0.001**
Without ECG	6.00	1.75 (5.00,6.75)		6.00	0.00 (6.00-6.00)	

Within ECG: Case management with ECG response, Without ECG: The ECG response given is considered correct and only case management, OSCE: cases obtained from objective structured clinical examination (OSCE) questions, PubMed: Cases obtained from case reports in PubMed IQR: Inter quartile ratio, CI: Confidence interval.

p < 0.05 was considered statistically significant.

* According to Mann-Whitney U test results.

Upon examining GPT-4’s responses for case management, no statistically significant difference was found between the management of PubMed and OSCE cases in the with ECG evaluation group (p = 0.457). Additionally, no statistically significant difference was found between the management of PubMed and OSCE cases in the without ECG group (p = 0.957). Detailed information is shown in Table 3.

**Table 3:** Comparison of Within ECG and Without ECG groups with Pubmed and OSCE case groups.

	Within ECG			Without ECG		
	Median	IQR (%25, %75)	P Value^*^	Median	IQR (%25, %75)	P Value^*^
OSCE	4.00	3.00 (3.00,6.00)	0.473	6.00	1.75 (5.00,6.75)	0.781
PubMed	5.00	3.00 (3.00,6.00)		6.00	0.00 (6.00-6.00)	

Within ECG: Case management with ECG response, Without ECG: The ECG response given is considered correct and only case management, OSCE: cases obtained from objective structured clinical examination (OSCE) questions, PubMed: Cases obtained from case reports in PubMed IQR: Inter quartile ratio, CI: Confidence interval.

p < 0.05 was considered statistically significant.

* According to Mann-Whitney U test results.

In evaluating GPT-4’s ECG interpretation for PubMed cases, it was found that GPT-4 provided correct (C = 9, 18%) and largely correct (LC = 12, 24%) responses for a total of 42% of the ECGs. Similarly, in the OSCE case group, it was found that GPT-4 could provide correct (C = 9 (18%)) and largely correct (LC = 10 (20%)) responses for a total of 38% of the ECGs. Detailed information is shown in Table 4.

**Table 4:** Evolution of ECG answer within group.

ECG Answers	Groups		Total
	OSCE	PubMed	
IC (n, %)	24 (%48.0)	19 (%38.0)	43 (%43.0)
LIC (n, %)	7 (%14.0)	10 (%20.0)	17 (%17.0)
LC (n, %)	10 (%20.0)	12 (%24.0)	22 (%22.0)
C (n, %)	9 (%18.0)	9 (%18.0)	18 (%18.0)
Total (n, %)	50 (%100.0)	50 (%100.0)	100 (%100.0)

IC: Incorrect answers, LIC: Largely incorrect answers, LC: Largely correct answers, C: Correct answers, PubMed: Total number of responses from reviewers in the PubMed group ECGs, OSCE: Total number of responses from reviewers in the OSCE group ECGs, Total: Total number of responses from reviewers in both group ECGs.

Regarding critical errors in ECG interpretations and case management provided by GPT-4, it was found that GPT-4 made critical errors in 46% of the ECG responses in the OSCE group and 50% in the PubMed group. No statistically significant difference was found between the two groups (p = 0.689). When examining critical errors in case management, it was found that 32% of the managements in the OSCE group and 14% in the PubMed group had critical errors. A statistically significant difference was found between the two groups (p = 0.032). Detailed information is shown in Table 5.

**Table 5:** Evaluation of evaluators’ answers to critical action questions.

	ECG		P value^*^	Case Management		P value^*^
	Yes	No		Yes	No	
PubMed (n, %)	25 (%50.0)	25 (%50.0)	0.689	7 (%14.0)	43 (%86.0)	0.032
OSCE (n, %)	23 (%46.0)	27 (%54.0)		16 (%32.0)	34 (%68.0)	
Total (n, %)	48 (%48.0)	52 (%52.0)		23 (%23.0)	77 (%77.0)	

* According to χ² tests results.

p < 0.05 was considered statistically significant.

PubMed: Total number of responses from reviewers in the PubMed group, OSCE: Total number of responses from reviewers in the OSCE group Total: Total number of responses from reviewers in both groups.

Lastly, the inter-rater reliability among the evaluators in our study indicated good agreement (ICC = 0.725, F = 3.72, p < 0.001).

## Discussion

Our study findings reveal that GPT-4, while demonstrating potential in ECG interpretation and case management, shows variable accuracy depending on the complexity of the cases. Notably, GPT-4’s overall performance in managing cases without ECG interpretation was statistically more successful compared to when ECG evaluation was included. A critical finding of our research is the substantial incidence of critical errors made by GPT-4 in both ECG interpretations and patient management. These errors are concerning, as they could potentially lead to severe adverse outcomes, including increased morbidity and mortality. The presence of significant differences in only a subset of cases also suggests that GPT-4’s performance varies depending on the ECG type. Simple ECGs—such as those showing normal sinus rhythm or isolated first-degree AV block—were interpreted with greater accuracy, whereas more complex arrhythmias (e.g., atrial flutter, SVT) or ECGs with subtle ischemic changes led to greater misinterpretation and critical management errors. This pattern indicates that GPT-4 currently performs better with textbook-like ECGs, while struggling with rhythm complexity and clinically nuanced presentations. These findings highlight the limitations of large language models in handling visually complex or borderline cases, and underscore the importance of further development before clinical deployment in high-risk settings.

ECG evaluation is crucial both during resuscitation and in patient management in emergency departments [22]. Emergency medicine, like many other medical specialties, has identified a variety of potential AI applications [23]. The usability of GPT-4 in ECG evaluation, considering the intensity of emergency departments, could greatly benefit emergency physicians during patient evaluations. In a study by Günay et al., it was found that GPT-4 could provide more accurate responses compared to emergency medicine specialists and cardiologists when ECG descriptions were provided. However, in this study, the actual ECGs were not used, only the descriptions [24]. In our study, it was found that GPT-4 provided incorrect or largely incorrect responses for more than half of the ECGs in both the OSCE and PubMed case groups. ECG interpretation requires analyzing complex visual patterns that may not be easily described by text alone. GPT-4, which is primarily a text-based model, might not yet have the capability to accurately interpret these visual patterns. We believe complexity of the visual data may be the reason of difference between Gunay et al. ‘ s sudy and our study.

ECG evaluation is crucial both during resuscitation and in patient management in emergency departments [22]. The usability of GPT-4 in ECG evaluation, considering the intensity of emergency departments, could greatly benefit emergency physicians during patient evaluations [23]. In recent years, AI-based decision support systems have started to be used in various aspects of cardiovascular diseases [24]. In a study by Günay et al., it was found that GPT-4 could provide more accurate responses compared to emergency medicine specialists and cardiologists when ECG descriptions were provided. However, in that study, the actual ECGs were not used, only the descriptions [25]. In our study, it was found that GPT-4 provided incorrect or largely incorrect responses for more than half of the ECGs in both the OSCE and PubMed case groups. Additionally, evaluators concluded that nearly half of the ECG interpretations had critical errors. When patient management was evaluated along with ECG interpretations, it was found that errors largely stemmed from GPT-4’s incorrect ECG interpretations. We believe the discrepancy between Günay et al.’s [25] study and our study can be attributed to the complexity of visual data. ECG interpretation requires analyzing complex visual patterns that may not be easily described by text alone. GPT-4, which is primarily a text-based model, might not yet have the capability to accurately interpret these visual patterns. Based on these results, we believe that using GPT-4 for ECG evaluation in emergency departments is not appropriate at this stage and could cause critical harm to patients.

The decision to use ChatGPT-4 for patient management in the emergency department requires careful consideration. While ChatGPT-4 has shown potential in various medical applications, including ECG interpretation and clinical decision support, its specific role in managing critically ill patients in the emergency department remains uncertain [26,27]. In our study, GPT-4 made critical errors in 32% of cases from the OSCE question book and 14% of cases from PubMed, which could potentially lead to morbidity and mortality in patients. When ECG evaluations were included in patient management, this error rate approached 50%. Given these findings, we do not recommend the use of GPT-4 for patient management in emergency departments, particularly for ECG evaluations. It appears that GPT-4 may cause significant errors in patient management in emergency departments at this stage.

Our study has several limitations. First, each case output was entered into GPT-4 once, and the responses were recorded. The consistency of the responses with repeated inputs was not evaluated. Second, the OSCE sample cases we referenced may have been accessed by GPT-4. This means it may have encountered these cases before. However, since the reference book primarily contains multiple-choice answers rather than patient management scenarios, we do not believe this affected GPT-4’s case evaluations. Another limitation of this study is the absence of a validated difficulty index for the OSCE cases, which may have helped further stratify case complexity and better contextualize GPT-4’s performance across varying clinical scenarios.

## Conclusions

In conclusion, our study found that the use of GPT-4 for ECG evaluation and subsequent patient management in emergency departments is not appropriate. The model demonstrated a propensity for significant errors that could potentially result in morbidity and mortality in patients.

## Supporting information

S1 Appendix Responses to ECG cases and case managements provided by GPT-4. (DOCX)

## References

[1] Garvey JL. ECG techniques and technologies. Emerg Med Clin North Am. 2006;24(1):209–25. doi: 10.1016/j.emc.2005.08.013 16308121

[2] Ose B, Sattar Z, Gupta A, Toquica C, Harvey C, Noheria A. Artificial intelligence interpretation of the electrocardiogram: a state-of-the-art review. Curr Cardiol Rep. 2024;26(6):561–80. 38753291 10.1007/s11886-024-02062-1

[3] Parsa S, Somani S, Dudum R, Jain SS, Rodriguez F. Artificial intelligence in cardiovascular disease prevention: is it ready for prime time?. Curr Atheroscler Rep. 2024;26(7):263–72. doi: 10.1007/s11883-024-01012-3 38780665 PMC11457745

[4] GPT-4. 2023. Accessed 2024 May 31. https://openai.com/index/gpt-4/.

[5] Fijačko N, Prosen G, Abella BS, Metličar Š, Štiglic G. Can novel multimodal chatbots such as bing chat enterprise, ChatGPT-4 Pro, and google bard correctly interpret electrocardiogram images?. Resuscitation. 2023;193:110009. doi: 10.1016/j.resuscitation.2023.110009 37884222

[6] Zhu L, Mou W, Wu K, Lai Y, Lin A, Yang T, et al. Multimodal ChatGPT-4V for electrocardiogram interpretation: promise and limitations. J Med Internet Res. 2024;26:e54607. doi: 10.2196/54607 38764297 PMC11237788

[7] Birkun A. Performance of an artificial intelligence-based chatbot when acting as EMS dispatcher in a cardiac arrest scenario. Intern Emerg Med. 2023;18(8):2449–52. doi: 10.1007/s11739-023-03399-1 37603142

[8] Birkun AA, Gautam A. Instructional support on first aid in choking by an artificial intelligence-powered chatbot. Am J Emerg Med. 2023;70:200–2. doi: 10.1016/j.ajem.2023.06.010 37330383

[9] Pham C, Govender R, Tehami S, Chavez S, Adepoju OE, Liaw W. ChatGPT’s performance in cardiac arrest and bradycardia simulations using the American Heart Association’s advanced cardiovascular life support guidelines: exploratory study. J Med Internet Res. 2024;26:e55037. 38648098 10.2196/55037 PMC11074885

[10] Newble D. Techniques for measuring clinical competence: objective structured clinical examinations. Med Educ. 2004;38(2):199–203. doi: 10.1111/j.1365-2923.2004.01755.x 14871390

[11] Minato H, Yoshikawa A, Tsuyama S, Katayanagi K, Hachiya S, Ohta K, et al. Fatal arrythmia in a young man after COVID-19 vaccination: an autopsy report. Medicine (Baltimore). 2024;103(5):e37196. 38306524 10.1097/MD.0000000000037196 PMC10843519

[12] Jokšić-Mazinjanin R, Marić N, Đuričin A, Bjelobrk M, Bjelić S, Trajković M. Simultaneous double-vessel coronary thrombosis with sudden cardiac arrest as the first manifestation of COVID-19. Medicina. 2023;60(1):39. 38256301 10.3390/medicina60010039 PMC10820554

[13] Alzeer AA, Suliman I, Altamimi M, Alshudukhi AM, Alzeer AA, Alwasidi EO. Acute myocardial infarction associated with amphetamine use and smoking in a young healthy individual. Cureus. 2023;15(12):e50323. doi: 10.7759/cureus.50323 38089954 PMC10712411

[14] Koo AY, Gao L. Post-return of spontaneous circulation (ROSC) accelerated idioventricular rhythm in the setting of severe pancreatitis and hyperkalemia. Cureus. 2022;14(3):e23573. 35495000 10.7759/cureus.23573 PMC9045550

[15] Talwar D, Kumar S, Acharya S, Khanna S, Hulkoti V. Paroxysmal supraventricular tachycardia and cardiac arrest: a presentation of pulmonary embolism with infarction as a sequela of long COVID syndrome. Cureus. 2021;13(10):e18572. 34765348 10.7759/cureus.18572 PMC8575334

[16] Verma A, Nanda V, Kabi A, Baid H. Marijuana-induced acute myocardial infarction in a young adult male. BMJ Case Rep. 2021;14(7):e243335. doi: 10.1136/bcr-2021-243335 34257123 PMC8278926

[17] Bracey A, Meyers HP, Smith SW. Post-arrest wide complex rhythm: What is the cause of death?. Am J Emerg Med. 2021;45:683.e5-683.e7. doi: 10.1016/j.ajem.2020.12.028 33353817

[18] Okonkwo ER, Schuetz C, Hyman B, Samuels B, Sayad D, Bower J. Electrocardiograms revealing epsilon waves following use of hormone supplements in young cardiac arrest patient. Cureus. 2021;13(4):e14305. 33968517 10.7759/cureus.14305 PMC8098703

[19] Liu L, Wang D. A rare transitory change of the De Winter ST/T-wave complex in a patient with cardiac arrest: a case report. Medicine (Baltimore). 2020;99(19):e20133. doi: 10.1097/MD.0000000000020133 32384494 PMC7220489

[20] Healey T, Buckley C 2nd, Mollman M. Motor vehicle collision secondary to ventricular dysrhythmia: a case report of Brugada syndrome. J Emerg Med. 2017;52(2):227–30. 27751697 10.1016/j.jemermed.2016.09.022

[21] Aldeen AZ, Rosenbaum DH. 1200 questions to help you pass the emergency medicine boards. 2nd ed. 2008.

[22] Lindsey S, Moran TP, Stauch MA, Lynch AL, Grabow Moore K. Bridging the gap: evaluation of an electrocardiogram curriculum for advanced practice clinicians. West J Emerg Med. 2024;25(2):155–9. doi: 10.5811/westjem.18085 38596911 PMC11000549

[23] Ahun E, Demir A, Yiğit Y, Tulgar YK, Doğan M, Thomas DT. Perceptions and concerns of emergency medicine practitioners about artificial intelligence in emergency triage management during the pandemic: a national survey-based study. Front Public Health. 2023;11:1285390. 37965502 10.3389/fpubh.2023.1285390 PMC10640989

[24] Günay S, Öztürk A, Yiğit Y. The accuracy of Gemini, GPT-4, and GPT-4o in ECG analysis: a comparison with cardiologists and emergency medicine specialists. Am J Emerg Med. 2024;84:68–73. doi: 10.1016/j.ajem.2024.07.043 39096711

[25] Günay S, Öztürk A, Özerol H, Yiğit Y, Erenler AK. Comparison of emergency medicine specialist, cardiologist, and chat-GPT in electrocardiography assessment. Am J Emerg Med. 2024;80:51–60. doi: 10.1016/j.ajem.2024.03.017 38507847

[26] Lisicic A, Serman A, Jordan A, Jurin I, Novak A, Benko I, et al. Does ChatGPT-4 succeed in the ECG interpretation: friend or foe to cardiologists?. Europace. 2024;26(Supplement_1). doi: 10.1093/europace/euae102.655

[27] Haim A, Katson M, Cohen-Shelly M, Peretz S, Aran D, Shelly S. Evaluating GPT-4 as a clinical decision support tool in ischemic stroke management. medRxiv. 2024;01. 10.2196/60391 PMC11928773 40053715

