# Supplementary material for: Evaluating GPT-4’s role in critical patient management in emergency departments
Source: PLoS One. 2025 Jul 24;20(7):e0327584. doi: 10.1371/journal.pone.0327584 (PMC12288989; doi:10.1371/journal.pone.0327584)
Supplement: S1 Appendix — (DOCX) [file pone.0327584.s001.docx]

Appendix. Responses to ECG cases and case managements provided by GPT-4

| OSCE | | | |
| --- | --- | --- | --- |
| CN | CASE | ECG answers | Case Management |
| 1 | 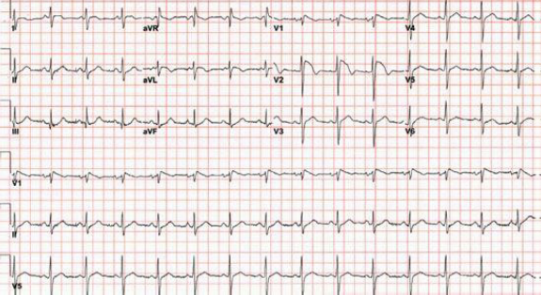  A 43-year-old otherwise healthy male presents with a 1-week history of a productive cough, subjective fevers and chills, and mild shortness of breath, without chest pain. The patient was transferred from an urgent care facility, where he was found to be hypoxic, with a SaO2 of 87% on room air (RA). | This is a 12-lead ECG tracing. Here's a systematic approach to interpret it:  1. Rate and Rhythm:  - The rhythm appears regular.  - To calculate the rate, you can count the number of large squares between two R waves and divide that into 300. This ECG seems to have about 3 large squares between R waves, suggesting a heart rate of around 100 beats per minute.  2. Axis:  - The axis can be determined by looking at leads I and aVF. This ECG shows a positive deflection in lead I and a positive deflection in aVF, suggesting a normal axis.  3. P waves:  - P waves are present with a consistent shape, indicating that the atria are being depolarized from the sinoatrial node.  4. PR Interval:  - The PR interval is the distance from the onset of the P wave to the start of the QRS complex. It appears to be within the normal range (0.12 to 0.20 seconds).  5. QRS Complex:  - The QRS complexes are narrow, suggesting that ventricular depolarization is occurring normally through the His-Purkinje system.  6. ST Segment and T wave:  - The ST segments appear to be at baseline without elevation or depression.  - T waves are positive in most leads except for aVR, which is normal.  7. QT Interval:  - The QT interval seems to be of normal duration.  Preliminary Diagnosis: The ECG suggests a regular sinus rhythm with a normal rate and no immediate abnormalities in the intervals or waveforms. However, this is a basic interpretation and further clinical correlation is necessary. Patient history, symptoms, and possibly further diagnostic tests would help in forming a complete diagnosis. | This patient presents with symptoms and signs suggestive of a respiratory infection, possibly pneumonia, given the productive cough, fevers, chills, and hypoxia. The normal ECG is reassuring but does not exclude a serious illness.  Here’s a step-by-step approach to manage this patient in the emergency department:  1. Airway Management: Ensure the patient has a patent airway. If the patient is talking in full sentences and not in respiratory distress, the airway is likely intact.  2. Breathing:  - Administer high-flow oxygen via a non-rebreather mask to address hypoxia immediately.  - Prepare for possible escalation to positive pressure ventilation if there is no improvement or if the patient deteriorates.  3. Circulation:  - Obtain intravenous (IV) access with at least one large-bore cannula.  - Monitor blood pressure and heart rate; administer IV fluids if there are signs of hypotension or shock.  - Consider drawing blood cultures before antibiotics if sepsis is suspected.  4. Disability:  - Evaluate the patient’s level of consciousness, using the AVPU scale (Alert, responds to Voice, responds to Pain, Unresponsive).  - Check blood glucose to rule out hypoglycemia as a contributing factor.  5. Exposure:  - Conduct a full examination, including auscultation of the chest. Look for signs of consolidation or other causes of hypoxia.  6. Investigations:  - Obtain a chest X-ray to look for evidence of pneumonia or other pathology.  - Complete blood count (CBC), comprehensive metabolic panel (CMP), lactate, and inflammatory markers such as C-reactive protein (CRP) or procalcitonin should be ordered.  - Consider arterial blood gas (ABG) analysis to assess the degree of hypoxia and acid-base status.  7. Treatment:  - If pneumonia is suspected, initiate broad-spectrum antibiotics empirically, covering both typical and atypical pathogens.  - Provide supportive care with antipyretics and analgesics as needed.  - Fluid resuscitation should be balanced against the risk of fluid overload, especially if the patient has signs of heart failure or other reasons for fluid retention.  8. Monitor and Reassess:  - Regularly reassess the patient’s vital signs, oxygen saturation, respiratory rate, and level of consciousness.  - Be prepared to escalate care if there is evidence of sepsis or respiratory failure.  9. Additional Considerations:  - Assess the need for additional supportive measures, such as bronchodilators if there is wheezing or a history of reactive airway disease.  - Consider the need for admission to a higher level of care, such as the intensive care unit, if the patient shows signs of respiratory failure or septic shock.  10. Prevention:  - Implement droplet precautions given the productive cough and potential for pneumonia.  11. Social and Follow-up:  - Arrange for social support if needed upon discharge.  - Ensure appropriate follow-up with primary care or a pulmonologist.  Always correlate clinical findings with investigations to guide treatment. It's important to tailor the management plan to the individual patient's needs and response to treatment. |
| 2 |   A 22-year-old man presents with palpitations. He reports no chest pain, shortness of breath, or lower extremity edema. He states that he “got really drunk” the night before. He denies any past medical history, family history, or illicit drug use. His examination is unremarkable except for irregular tachycardia. | This ECG shows the following features:  1. Rate: The rate appears to be elevated; by quick estimation, it seems to be tachycardic, likely over 100 beats per minute.  2. Rhythm: The rhythm appears irregularly irregular, with no apparent repeating pattern.  3. P Waves: P waves are not clearly discernible, indicating that atrial activity is not organized or is occurring at a rate that is too fast to produce distinct P waves.  4. PR Interval: Cannot be measured due to the absence of discernible P waves.  5. QRS Complex: The QRS complexes appear narrow, suggesting that ventricular depolarization is occurring normally through the His-Purkinje system.  6. ST Segment and T wave: Variations in baseline and T wave morphology can be observed, likely due to the irregular rhythm.  7. QT Interval: Variable, due to the irregular rhythm.  Preliminary Diagnosis: The ECG is suggestive of atrial fibrillation (AF) with a rapid ventricular response. This is characterized by an irregularly irregular rhythm, absence of distinct P waves, and a narrow QRS complex. AF with rapid ventricular response is a common cardiac arrhythmia and can be associated with a variety of conditions including but not limited to heart failure, hypertension, and hyperthyroidism.  The patient should be further assessed for the severity of symptoms, potential underlying causes, and risk of stroke. Management may include rate control with beta-blockers or calcium channel blockers, rhythm control with antiarrhythmic drugs, and anticoagulation therapy to prevent thromboembolic events, based on clinical guidelines and the patient’s individual risk factors. | Given the patient's presentation with palpitations and an ECG suggestive of atrial fibrillation (AF) with rapid ventricular response, and in the context of a recent history of heavy alcohol consumption, the most appropriate management steps include:  1. Stabilization: Ensure the patient is stable with a patent airway, breathing adequately, and has sufficient circulation. Continuous monitoring of vital signs is crucial.  2. Oxygen and IV Access: Administer oxygen if the patient is hypoxic and establish intravenous access for potential medications and fluid administration.  3. Rate Control: Considering his age and hemodynamic stability, rate control can be achieved with beta-blockers like metoprolol or non-dihydropyridine calcium channel blockers like diltiazem, provided there are no contraindications.  4. Rhythm Control: As this may be the first episode of AF and related to alcohol intake, known as "holiday heart syndrome," the decision for immediate rhythm control should be based on the duration of AF (if known), symptom severity, and underlying structural heart disease (which appears absent in this patient). Cardioversion is usually not the first line unless the patient is hemodynamically unstable.  5. Thromboembolic Risk Assessment: Since he is young and has no risk factors, the need for anticoagulation is low, but a formal risk assessment using tools like the CHA2DS2-VASc score should be done to guide therapy.  6. Labs and Imaging: Order a complete blood count, thyroid function tests, basic metabolic panel, and possibly a toxicology screen given the history of alcohol consumption. An echocardiogram may also be considered to evaluate for underlying structural heart disease.  7. Observation and Cardiology Consultation: Admit the patient for telemetry monitoring and cardiology consultation for further evaluation and management of the AF, including decisions regarding the need for rhythm control strategies like antiarrhythmic drugs or elective cardioversion, and the potential need for anticoagulation.  8. Education and Lifestyle Modification: Counsel the patient on the effects of alcohol on the heart and the importance of moderating intake to prevent future episodes. Discuss the nature of AF and the importance of follow-up.  9. Discharge Planning: If the patient returns to sinus rhythm and is stable, plan for outpatient follow-up, ensuring that he understands when to return to the ER (if symptoms recur or worsen) and has a follow-up appointment with cardiology.  10. Documentation: Document the episode, management, and the patient's response to therapy in his medical records for future reference.  The key with this patient is observation and ensuring stability, as many cases of "holiday heart syndrome" resolve on their own once the precipitating factor (in this case, alcohol) is removed. However, the potential for recurrence and the need for longer-term management should be assessed during follow-up. |
| 3 |   A 22-year-old man presents with an episode of left-sided chest pain that lasted 4 hours, but resolved an hour before presenting to the ED. The pain was dull and radiated to his left shoulder. The patient states that he used cocaine at a party 12 hours ago. Physical examination is unremarkable and vital signs are normal. | This ECG shows the following characteristics:  1. Rate: The heart rate appears to be slow; there are less than 60 beats per minute by the count of QRS complexes.  2. Rhythm: The rhythm looks regular.  3. P Waves: There are P waves present with a consistent morphology, indicating normal atrial depolarization.  4. PR Interval: The PR interval appears to be prolonged, exceeding one large square on the ECG paper, which suggests a value greater than 0.20 seconds.  5. QRS Complex: The QRS complexes appear to be narrow, indicating normal ventricular depolarization.  6. ST Segment and T wave: No significant ST segment elevations or depressions are seen, and the T waves are consistent in morphology in consecutive beats.  7. QT Interval: The QT interval is difficult to interpret without precise measurement, but it doesn't appear grossly abnormal.  Preliminary Diagnosis: The ECG demonstrates a bradycardia with a first-degree atrioventricular (AV) block, evidenced by a prolonged PR interval on a regular rhythm with a rate less than 60 beats per minute. This may be seen in well-trained athletes or as a result of increased vagal tone, but it could also be due to intrinsic cardiac abnormalities, certain medications, or electrolyte imbalances. Further clinical correlation and investigation are warranted to determine the etiology of the first-degree AV block and whether any intervention is necessary. | The presentation of chest pain in a young patient with recent cocaine use raises concern for cocaine-induced cardiac ischemia or infarction. The ECG findings suggest a first-degree atrioventricular (AV) block, which in itself is typically not an emergency. However, the clinical context is critical here, and the presence of chest pain in the context of cocaine use requires a thorough workup for potential acute coronary syndrome (ACS) even if the pain has resolved and the ECG does not show acute ischemic changes.  Here is an approach to manage this patient:  1. Stabilization: Ensure the patient is stable with continuous monitoring of vital signs and cardiac rhythm.  2. IV Access and Labs: Obtain IV access and send blood work, including complete blood count (CBC), basic metabolic panel (BMP), cardiac enzymes (troponins), and urine toxicology screen to confirm cocaine use.  3. Chest Pain Protocol: Follow the chest pain protocol which typically includes administration of aspirin (unless contraindicated), and considering nitroglycerin if hypertension is present. Beta-blockers are generally avoided in cocaine-induced chest pain due to the risk of unopposed alpha-adrenergic coronary vasoconstriction.  4. Risk Stratification: Perform risk stratification for ACS using clinical tools and judgement. Despite the normal vital signs and resolved symptoms, the history of cocaine use is concerning.  5. Echocardiogram: An urgent echocardiogram may be warranted to assess for wall motion abnormalities indicative of ischemia or infarction.  6. Observation and Monitoring: Admit the patient to a monitored setting, like an observation unit or telemetry floor, for continuous cardiac monitoring and repeated troponin measurements.  7. Cardiology Consultation: A cardiology consult should be obtained for further management which may include stress testing, coronary angiography, or other imaging as indicated.  8. Substance Use Counseling: The patient should receive counseling about the risks of cocaine use, including its potential to cause serious cardiovascular events. A referral to substance use disorder services and counseling should be made.  9. Documentation: Document the patient's presentation, management, and response to interventions in his medical records for future reference.  10. Follow-up: Arrange appropriate follow-up with both cardiology and substance use disorder services.  11. Education: Educate the patient on the signs and symptoms of ACS and the importance of seeking immediate medical attention if they recur.  It’s important to consider other causes of chest pain such as pneumothorax, musculoskeletal pain, and pericarditis in differential diagnosis, but the priority is to rule out life-threatening cardiac causes first. |
| 4 |   A 56-year-old man with long-standing hypertension and mild renal insufficiency presents to the ED with vague complaints of fatigue and generalized malaise. | This ECG displays several distinct characteristics:  1. Rate: The heart rate is rapid; by a quick estimation, it's well over 100 beats per minute.  2. Rhythm: The rhythm appears irregular with varying R-R intervals, which suggests an irregularly irregular rhythm.  3. P Waves: P waves are not discernible, suggesting that the atria are not depolarizing in a normal, organized fashion.  4. QRS Complex: The QRS complexes appear narrow, indicating that ventricular depolarization is likely occurring via the normal conduction system.  5. ST Segment: No significant ST segment elevation or depression can be discerned from this tracing.  6. T Waves: T wave morphology is difficult to assess due to the rapid rate, but no gross abnormalities are evident.  Preliminary Diagnosis: The ECG suggests atrial fibrillation with a rapid ventricular response, characterized by an irregularly irregular rhythm, absent P waves, and a rapid rate with narrow QRS complexes. This is a common arrhythmia that can be caused by a variety of factors including structural heart disease, electrolyte imbalances, and stimulant use, among others. The management would focus on rate control, stroke risk assessment, and investigation of underlying causes. | For this 56-year-old man with a history of hypertension and mild renal insufficiency who presents with fatigue and generalized malaise, and an ECG showing atrial fibrillation with a rapid ventricular response, a systematic approach to management should be considered:  1. Initial Assessment: Evaluate the patient’s airway, breathing, and circulation. Obtain vital signs to check for any instability, such as hypotension or tachycardia.  2. Oxygen and IV Access: Provide supplemental oxygen if the patient is hypoxic and establish IV access for medications and fluid administration if necessary.  3. Focused History and Physical Examination: While the ECG findings provide valuable information, it's important to conduct a thorough history and physical examination to determine the potential causes of his symptoms and to assess for any signs of heart failure or other organ dysfunction.  4. Laboratory Tests:  - Obtain a complete blood count, comprehensive metabolic panel (including electrolytes, renal function, and calcium), liver function tests, thyroid function tests, and coagulation profile.  - Check cardiac markers like troponins to rule out myocardial infarction, which can present with vague symptoms in the setting of AF.  5. Rate Control: Initiate rate control with medications such as beta-blockers or non-dihydropyridine calcium channel blockers. Be cautious with drug dosing given the patient's renal insufficiency.  6. Rhythm Control: Since this may not be the first episode of AF, a decision on rhythm control, which might include the use of antiarrhythmic medications, should be made in consultation with a cardiologist.  7. Anticoagulation: Evaluate the patient’s risk for thromboembolic events using the CHA2DS2-VASc score and consider anticoagulation accordingly. This should be balanced against any bleeding risks.  8. Echocardiography: Order an echocardiogram to assess the heart's structure and function, which can provide clues about the etiology of atrial fibrillation and evaluate for signs of heart failure.  9. Management of Comorbid Conditions: Adjust medications for hypertension and ensure that renal function is not further compromised. It may be necessary to modify dosages in the setting of renal insufficiency.  10. Monitoring and Admission: The patient should be placed on telemetry for continuous cardiac monitoring. Consider admitting the patient for further workup and management, especially if he is hemodynamically unstable, symptomatic, or if there is a concern for acute coronary syndrome or heart failure.  11. Counseling and Education: Explain the diagnosis to the patient, discuss the potential causes and the importance of follow-up. Educate about the signs and symptoms that should prompt immediate medical attention.  12. Follow-up: Ensure appropriate follow-up with a primary care physician or a cardiologist and adjust his long-term medications to better control his blood pressure and address his atrial fibrillation.  This patient's management would need to be individualized, particularly considering his renal insufficiency which can complicate the choice of medications for rate or rhythm control, and for anticoagulation. |
| 5 |   A 71-year-old female with a history of chronic obstructive pulmonary disease not dependent on home oxygen is brought in by EMS with a chief complaint of shortness of breath. Her family states that she seemed to get suddenly short of breath and diaphoretic at home after eating breakfast. Though the patient states her symptoms quickly resolved, her family was concerned because something “didn’t seem right” after watching her for a few hours. She denies any chest pain. In the ED, her vitals are T 97.9, P 105, BP 136/78, RR 22, SaO2 94% on RA. Her family states that her saturation is at her baseline. Her lung examination reveals minimal wheezes, though with diffusely diminished air exchange. A subsequent chest x-ray is negative. Her cardiac Troponin I (cTnI) results at 0.91 (normal 0.50). | This ECG shows the following features:  1. Rate: The rate seems to be within normal limits, roughly estimated between 60-100 beats per minute.  2. Rhythm: The rhythm appears regular with consistent R-R intervals.  3. P Waves: There are P waves before each QRS complex, indicating sinus rhythm.  4. PR Interval: The PR interval appears to be normal, consistent, and within the normal range of 0.12-0.20 seconds.  5. QRS Complex: The QRS complexes are narrow, suggesting normal ventricular depolarization through the His-Purkinje system.  6. ST Segment: The ST segments look to be at baseline without obvious elevation or depression.  7. T Waves: The T waves appear normal and upright in most leads, except in aVR where an inverted T wave is normal.  8. QT Interval: The QT interval seems to be within normal limits.  Preliminary Diagnosis: This ECG suggests a normal sinus rhythm without any obvious acute ST-T wave changes indicative of ischemia. There are no signs of hypertrophy or bundle branch blocks, and the rate and rhythm are within normal limits. This is a relatively normal ECG, and any symptoms or clinical concerns this patient has are not likely to be related to an acute cardiac event based on this ECG alone. Further clinical correlation is necessary, and if the patient is symptomatic, further investigations might be warranted based on the symptoms and clinical judgement. | This 71-year-old female with a history of COPD presents with acute onset of shortness of breath that has resolved, but with an elevated cardiac troponin I level. The described ECG shows normal sinus rhythm with no acute changes, and her chest X-ray is negative. The elevated troponin, even without chest pain or ECG changes, raises the concern for a possible cardiac event, such as a non-ST elevation myocardial infarction (NSTEMI), especially given her age and known COPD.  Management should include:  1. Airway and Breathing:  - Monitor respiratory status closely, given her history of COPD and acute symptoms.  - Administer supplemental oxygen to keep SaO2 >94%.  2. Circulation and Monitoring:  - Continue monitoring vital signs and cardiac rhythm.  - Start an IV line for possible medications and fluid administration.  3. Serial Troponins and ECGs:  - Repeat the troponin measurement and ECG in a serial fashion to look for dynamic changes.  - Consider ordering an NT-proBNP test to evaluate for heart failure.  4. Additional Labs and Imaging:  - Order a complete blood count, basic metabolic panel, B-type natriuretic peptide (BNP), D-dimer, and arterial blood gas if indicated by the respiratory status.  - Obtain a chest CT scan if there is a high clinical suspicion for pulmonary embolism, given the acute onset of symptoms and her respiratory disease.  5. Cardiology Consultation:  - Involve cardiology early for the elevated troponin and discuss the need for further workup, including stress testing, echocardiography, or coronary angiography.  6. Management of COPD:  - Treat any potential COPD exacerbation with bronchodilators, corticosteroids, and possibly antibiotics if an infectious component is suspected.  7. Risk Stratification:  - Assess for risk factors of coronary artery disease and for complications related to COPD.  8. Observation or Admission:  - Due to the troponin elevation and recent acute event, admit the patient for further monitoring and management, preferably in a setting where she can be observed closely, such as a telemetry unit.  9. Further Evaluation:  - Evaluate other potential causes of her symptoms, such as a pulmonary embolism or aortic dissection, which could cause shortness of breath and an elevated troponin without chest pain.  10. Patient and Family Education:  - Keep the patient and family informed about the findings, the need for further testing, and the potential causes of her symptoms.  11. Document:  - Ensure thorough documentation of the patient's presentation, clinical findings, management decisions, and the rationale for these decisions.  This patient's presentation could be multifactorial, and while the current symptoms have resolved, the elevated troponin is a significant finding that necessitates a comprehensive approach to her care. |
| 6 |   A 35-year-old woman presents with severe weakness, lightheadedness, and chest pain. Her blood pressure is 70/40 and her pulses are weak. | Based on the visual assessment of the ECG, it exhibits the following features:  1. Rate: The rate is fast, likely above 100 beats per minute given the number of QRS complexes in a short span on the paper.  2. Rhythm: It appears to be regular with consistent R-R intervals.  3. P Waves: P waves are not easily discernible, which may suggest an abnormal atrial rhythm or activity that is too fast to resolve separate P waves.  4. PR Interval: Cannot be determined due to the absence of identifiable P waves.  5. QRS Complex: The QRS complexes are narrow, suggesting that ventricular depolarization is proceeding normally via the His-Purkinje system.  6. ST Segment and T wave: The rapid rate makes assessment of the ST segment and T wave difficult, but there are no immediately obvious gross abnormalities.  7. QT Interval: The QT interval is difficult to evaluate accurately without distinct T waves and due to the tachycardia.  Preliminary Diagnosis: The ECG suggests a supraventricular tachycardia (SVT), which is a broad term that includes various conditions where the rhythm originates above the ventricles and causes a rapid heart rate. This could include atrial flutter, paroxysmal SVT, or other forms of tachyarrhythmias. Given the rapid rate and narrow QRS complexes, atrial flutter with a regular conduction to the ventricles is a possibility, but without distinct flutter waves, other forms of SVT cannot be excluded.  Clinical correlation is needed to determine the exact nature of the tachycardia, and management will depend on the patient's hemodynamic stability and symptoms. If the patient is stable, vagal maneuvers or pharmacologic interventions such as adenosine can be considered to slow the heart rate and possibly convert the rhythm to normal sinus. If the patient is unstable, synchronized cardioversion may be necessary. | This patient is presenting with hemodynamic instability, characterized by hypotension and symptoms of severe weakness, lightheadedness, and chest pain. The ECG indicates a supraventricular tachycardia (SVT), which could be contributing to her unstable state. Immediate management is critical and should follow the ACLS (Advanced Cardiac Life Support) guidelines for unstable tachycardia:  1. Immediate Actions:  - Attach the patient to a cardiac monitor, establish IV access, and start continuous pulse oximetry.  - Administer high-flow oxygen via a non-rebreather mask.  - Prepare for immediate resuscitation, including obtaining airway equipment and a defibrillator.  2. Hemodynamic Support:  - Initiate rapid IV fluid boluses to address hypotension.  - If the patient does not respond to fluids and remains hypotensive, consider the use of vasopressors such as norepinephrine or epinephrine.  3. Cardioversion:  - If the patient remains hemodynamically unstable despite initial resuscitation efforts, synchronized cardioversion should be performed promptly.  - Administer sedation if the patient is conscious and time allows, but do not delay cardioversion in a critically unstable patient.  4. Medications:  - Antiarrhythmic drugs can be considered if cardioversion is not immediately available or after cardioversion if SVT recurs.  5. Further Evaluation:  - Once stabilized, further workup is necessary to understand the underlying cause of her tachycardia and hypotension.  - This would include blood work to assess for myocardial infarction, electrolyte imbalances, complete blood count, and thyroid function tests.  6. Consultation and Admission:  - Immediate consultation with a cardiologist is warranted.  - The patient should be admitted to the ICU for close monitoring and further management, given her initial presentation with instability.  7. Follow-Up:  - If the rhythm is converted successfully, further evaluation to determine the cause of her SVT is essential.  - An echocardiogram should be obtained to assess for structural heart disease.  - In the case of recurrent SVT, she may need further electrophysiological testing or ablation therapy.  8. Documentation:  - Document all clinical findings, treatments administered, and the patient’s response to the interventions.  This patient's condition is potentially life-threatening and warrants immediate intervention to restore hemodynamic stability and normal cardiac rhythm. |
| 7 |   A 54-year-old man presents with a 4-hour history of chest pain, which has resolved at presentation. He also complains of diaphoresis, shortness of breath, and orthopnea. Vital signs are 98.6, 60, 22, 97/60, 92% on RA. Physical examination demonstrates an S3 and bilateral crackles to the midlung fields. | This ECG reveals the following features:  1. Rate: The heart rate appears to be normal to slightly elevated, which we could roughly estimate between 60 to 100 beats per minute.  2. Rhythm: There is a regular rhythm present with consistent R-R intervals.  3. P Waves: There is a P wave preceding each QRS complex, suggesting a sinus rhythm.  4. PR Interval: The PR interval appears to be within normal limits, consistent from beat to beat, and less than 0.20 seconds.  5. QRS Complex: The QRS complexes appear narrow, indicating a normal ventricular depolarization through the His-Purkinje system.  6. ST Segment: There are no obvious ST segment elevations or depressions on this tracing.  7. T Wave: T waves are present and appear normal in most leads.  8. QT Interval: The QT interval appears to be within normal limits, but exact measurement would be required for precise assessment.  Preliminary Diagnosis: This ECG suggests a normal sinus rhythm with no significant ST-T wave changes indicative of ischemia or infarction, and no immediate evidence of other abnormalities such as hypertrophy or bundle branch blocks. It appears to be a relatively normal ECG. However, ECG findings should always be interpreted in the context of the patient's clinical presentation, and further diagnostic workup should be based on the patient’s symptoms and history. | The patient's presentation suggests a possible acute cardiac event, despite a normal ECG. The symptoms of chest pain, diaphoresis, shortness of breath, and orthopnea, combined with an S3 gallop and bilateral crackles on lung auscultation, are concerning for heart failure, possibly secondary to ischemia.  Here is how to manage this patient:  1. Immediate Care:  - Place the patient on continuous cardiac monitoring and pulse oximetry.  - Administer supplemental oxygen to keep SaO2 >94%.  2. IV Access and Fluids:  - Establish IV access.  - Given the low blood pressure, caution is needed with fluid management. Fluids may be necessary if the patient is hypovolemic, but in the case of pulmonary edema, which the crackles may suggest, fluids should be avoided or used sparingly.  3. Hemodynamic Support:  - Since the blood pressure is on the lower side, vasopressors may be required if the patient does not respond to initial treatment and remains hypotensive.  4. Pharmacologic Therapy:  - If heart failure is suspected, consider the use of medications such as diuretics (e.g., furosemide) to reduce fluid overload.  - Nitroglycerin may help with both chest pain and preload reduction if the blood pressure allows.  5. Further Assessment:  - A chest X-ray is necessary to look for signs of heart failure or other lung pathology.  - Blood tests should be ordered, including cardiac enzymes (troponins), complete blood count, basic metabolic panel, liver function tests, coagulation profile, and B-type natriuretic peptide (BNP).  - Repeat ECGs and serial troponin measurements should be obtained to monitor for any changes indicative of myocardial infarction.  6. Echocardiogram:  - An urgent echocardiogram can assess left ventricular function and rule out other causes of acute heart failure or cardiogenic shock.  7. Specialist Consultation:  - Consult cardiology for further assessment and management. This might include considerations for advanced therapies or interventions, like coronary angiography.  8. Intensive Care:  - The patient should be admitted to the intensive care unit for close monitoring and management given the potential for acute coronary syndrome and acute heart failure.  9. Patient Education:  - Explain all interventions and the importance of immediate and continuous monitoring due to the risk of deterioration.  10. Document:  - Ensure all findings, interventions, and patient responses are well documented.  The management plan may need to be adjusted as further information is obtained, but initial stabilization, assessment, and treatment should not be delayed. |
| 8 |   A 65-year-old man presents with a syncopal event without prodromal symptoms. His vital signs are 98.6, 60, 18, 142/75, 99% RA. | This ECG reveals the following key features:  1. Rate: The heart rate seems to be within normal limits, which is generally 60-100 beats per minute.  2. Rhythm: The rhythm appears to be regular with consistent R-R intervals.  3. P Waves: P waves are visible and appear normal, with one P wave preceding each QRS complex, which suggests a sinus rhythm.  4. PR Interval: The PR interval appears to be normal, less than 0.20 seconds.  5. QRS Complex: The QRS complexes are narrow, suggesting normal ventricular depolarization.  6. ST Segment: There are no obvious ST segment elevations or depressions visible.  7. T Waves: The T waves appear upright and normal in most leads, without significant inversion or flattening.  8. QT Interval: The QT interval appears to be within normal limits.  Preliminary Diagnosis: The ECG suggests a normal sinus rhythm with no acute ST-T changes. There is no evidence of acute ischemia, significant arrhythmias, or other abnormalities such as bundle branch block or atrial enlargement on this tracing. However, ECG interpretation should always be correlated with the patient's clinical presentation, and additional diagnostic workup should be considered based on symptoms and clinical history. | A syncopal event without warning in a 65-year-old man is concerning and warrants a thorough evaluation. The ECG, as described, is normal, but syncope can be a sign of underlying cardiac issues that may not be evident on a resting ECG.  Here's how to proceed with his management:  1. Immediate Care: Place the patient on continuous cardiac monitoring to detect any transient arrhythmias, establish IV access, and initiate continuous pulse oximetry.  2. Post-Syncopal Evaluation:  - Conduct a detailed history of the events surrounding the syncope, including any possible triggers, duration, and recovery.  - Perform a thorough physical examination with a focus on neurological and cardiovascular assessment.  3. Laboratory Tests:  - Order a complete blood count, basic metabolic panel, cardiac enzymes, and coagulation studies to rule out metabolic causes, myocardial infarction, or a hematologic issue.  - Check orthostatic vital signs to see if there is a significant drop in blood pressure upon standing, which can indicate orthostatic hypotension.  4. Cardiac Imaging and Monitoring:  - Obtain an echocardiogram to assess for structural heart disease.  - Consider a 24-48 hour Holter monitor or a longer event monitor to evaluate for intermittent arrhythmias that could have led to the syncopal event.  5. Additional Diagnostics:  - If initial evaluations are non-diagnostic, consider further testing such as carotid sinus massage (in a controlled environment), tilt-table testing, or electrophysiological studies to investigate for other causes of syncope.  6. Specialist Referral:  - A cardiology consultation may be valuable for further evaluation, especially if the initial workup suggests a cardiac cause.  - If no cardiac cause is identified, a neurology consultation could be helpful if a neurological cause of syncope is suspected.  7. Observation:  - Depending on the initial findings, admit the patient for observation, especially if there are concerns for a cardiac etiology or if the cause of syncope remains unclear.  8. Review Medications:  - Review the patient's current medications for any that may cause or contribute to syncope, such as antihypertensives or diuretics.  9. Patient Education:  - Educate the patient about the need for further testing and ensure they understand the importance of follow-up appointments.  10. Documentation:  - Document the event, clinical findings, management decisions, and patient education thoroughly.  Syncope can have many causes, and a normal ECG does not rule out serious underlying conditions. An individualized approach based on the patient's medical history, medications, and results of initial testing is essential. |
| 9 |   A 65-year-old woman presents with lightheadedness. She denies chest pain or shortness of breath. Vital signs are 99.0°F, 160, 20, 144/75, 96% RA. | This ECG presents with the following characteristics:  1. Rate: The heart rate is rapid.  2. Rhythm: The rhythm is regular with no clear P waves visible, indicating a supraventricular origin.  3. QRS Complex: The QRS complexes appear narrow, which typically suggests that the electrical impulse is traveling through the normal conduction pathway within the ventricles.  4. P Waves: P waves are not discernible, which can occur in certain tachyarrhythmias.  5. ST Segment and T Waves: ST segments and T waves are difficult to evaluate due to the tachycardia.  6. Overall Impression: The tracing shows a sawtooth-like pattern in the inferior leads (II, III, and aVF) and V1, which is characteristic of atrial flutter.  Preliminary Diagnosis: Based on the ECG findings described, the most likely diagnosis is atrial flutter with a rapid ventricular response. Atrial flutter is an arrhythmia caused by a reentrant circuit in the atrium, leading to rapid contractions of the atrium at a rate of around 300 beats per minute, with a variable degree of conduction block at the AV node, which can lead to a regular ventricular rate as seen here.  Management should be guided by the patient's hemodynamic stability. If the patient is unstable, immediate synchronized cardioversion is indicated. If stable, rate control with medications such as beta-blockers or calcium channel blockers and anticoagulation should be considered, and a cardiology consultation should be obtained for potential rhythm control strategies. | The patient is presenting with lightheadedness and a rapid heart rate of 160 bpm. The ECG description indicates atrial flutter with a rapid ventricular response. Here's how to proceed with her management:  1. Initial Assessment:  - Place the patient on continuous cardiac monitoring.  - Attach pulse oximetry and administer oxygen if necessary.  - Establish IV access for medications and fluid administration.  2. Stabilization:  - Assess her hemodynamic stability. If she is unstable (e.g., hypotension, altered mental status, signs of shock), immediate synchronized cardioversion is indicated.  3. Rate Control:  - If the patient is stable, initiate rate control with medications. Beta-blockers or calcium channel blockers are commonly used to control ventricular rate.  - Given her age and the rapid heart rate, these medications should be used with caution and titrated to the patient's response.  4. Further Evaluation:  - Order laboratory tests including a complete blood count, basic metabolic panel, cardiac enzymes, thyroid function tests, and coagulation studies to assess for underlying causes.  - Obtain a chest X-ray to evaluate for other potential causes of her symptoms.  - An echocardiogram should be performed to assess for structural heart disease.  5. Anticoagulation:  - Assess for stroke risk using the CHA2DS2-VASc score and initiate anticoagulation if appropriate.  6. Consultation:  - Consult cardiology for further management, which may include consideration for rhythm control strategies like electrical or chemical cardioversion, or longer-term management with ablation.  7. Observation:  - Admit the patient for telemetry monitoring and further management, particularly given the new onset of rapid atrial flutter.  8. Patient Education:  - Educate the patient on the importance of follow-up, medication adherence, and monitoring for any recurrent symptoms.  9. Documentation:  - Document the patient's symptoms, clinical findings, ECG interpretation, and the rationale behind the management plan.  This patient requires close monitoring and a thorough workup to identify the cause of her lightheadedness and to manage the atrial flutter appropriately. |
| 10 |   A 55-year-old man without any past medical history presents with chest pain. Physical examination is normal. | The ECG you've provided shows several notable features:  1. Rate: The heart rate is rapid, which seems to be above 100 beats per minute as estimated by the number of QRS complexes.  2. Rhythm: The rhythm appears to be regular with consistent R-R intervals, suggesting a supraventricular source.  3. P Waves: P waves are not clearly discernible, indicating an abnormal atrial rhythm or activity too fast to result in distinct P waves, or they are hidden within the QRS complexes.  4. PR Interval: Due to the absence of visible P waves, the PR interval cannot be measured.  5. QRS Complex: The QRS complexes appear narrow, which generally suggests that the ventricles are being depolarized through the normal conduction pathway.  6. ST Segment and T Waves: Due to the rapid heart rate, it's difficult to evaluate the ST segment and T waves thoroughly; however, there don't seem to be any significant ST elevations or depressions on first glance.  7. Overall Impression: The ECG presents with a regular rhythm and a narrow QRS complex at a high rate, which is most consistent with a diagnosis of supraventricular tachycardia (SVT).  Preliminary Diagnosis: Given the ECG findings described, the most likely diagnosis is supraventricular tachycardia (SVT). SVT is an umbrella term for several tachycardia rhythms that originate above the ventricular tissue.  Management would depend on the patient's clinical status. If the patient is hemodynamically stable, vagal maneuvers followed by pharmacologic interventions, such as adenosine, may be attempted. If the patient is unstable or does not respond to medical therapy, synchronized cardioversion may be necessary. The presence of an SVT also prompts an investigation into the underlying cause, which can include but is not limited to, electrolyte imbalances, thyroid disease, and heart disease. | A 55-year-old man with no past medical history presenting with chest pain and an ECG consistent with supraventricular tachycardia (SVT) requires immediate and careful management. Even if the physical examination is normal, chest pain in a person of this age warrants a cautious approach due to the potential for underlying ischemic heart disease. Here's how to manage the situation:  1. Cardiac Monitoring: Place the patient on continuous cardiac monitoring to watch for changes in heart rate and rhythm.  2. IV Access and Labs:  - Establish IV access.  - Order labs including cardiac troponins to rule out myocardial infarction, a complete blood count, basic metabolic panel, coagulation studies, and thyroid function tests.  3. Oxygen and Pain Management:  - Administer supplemental oxygen if oxygen saturation is below 94%.  - Provide analgesia to manage chest pain, ensuring not to use medications that may adversely affect SVT.  4. Rate Control:  - If the patient is hemodynamically stable, attempt vagal maneuvers first.  - If vagal maneuvers do not work, consider administering adenosine if there are no contraindications. Adenosine can temporarily halt conduction through the AV node and may terminate the SVT.  5. ECG Interpretation:  - If the rhythm does not convert with initial management, a second ECG should be obtained to reassess the rhythm and look for ischemic changes.  6. Cardiology Consultation:  - Regardless of whether the SVT resolves, the patient should have a cardiology consultation for further evaluation, given the presentation with chest pain.  7. Chest Pain Protocol:  - Follow a chest pain protocol which might include nitroglycerin, aspirin, and potentially other antianginal therapies, after ruling out contraindications such as right ventricular infarction or severe aortic stenosis.  8. Imaging:  - Obtain a chest X-ray to look for other possible causes of chest pain.  - Consider further imaging such as a CT angiogram if a pulmonary embolism is suspected or coronary angiography if ischemic heart disease is a concern.  9. Admission:  - Given the presentation with chest pain, admit the patient for observation and further workup, including possible stress testing, echocardiography, or coronary angiography.  10. Patient Education and Follow-Up:  - Explain the findings and management plan to the patient.  - Ensure that the patient understands the need for further testing and observation.  11. Documentation:  - Document all clinical findings, the patient’s response to interventions, and the management plan thoroughly.  Chest pain in this context must be taken seriously, and while SVT is a significant finding, it is crucial not to overlook potential acute coronary syndrome or other life-threatening conditions. |
| PubMed** | | | |
| 1 | 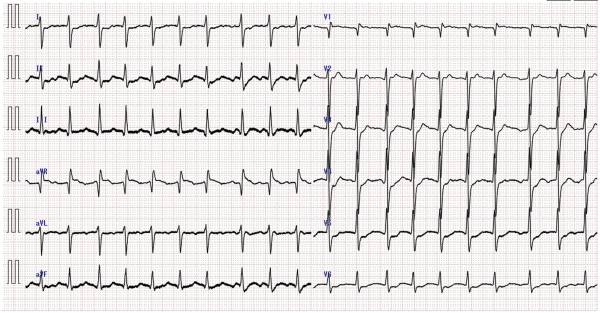  A Japanese man developed a fever a day after receiving the second dose of the COVID-19 vaccine (Pfizer-BioNTech COVID-19 mRNA vaccine). The following day, he suddenly lost consciousness and was rushed to the emergency room. He had a history of atopic dermatitis in childhood but no other health issues. Electrocardiography showed ventricular tachycardia transitioning to pulseless electrical activity with defibrillation. Resuscitation efforts resumed spontaneous heartbeat; however, the patient did not regain consciousness. When the heartbeat improves, the electrocardiography image is as above. | This ECG shows a normal sinus rhythm with a rate that appears to be within normal limits (we can't calculate the exact rate without the paper speed or calibration, but a rough estimate can be done by eye). There is a consistent P wave before each QRS complex, and the PR interval is normal, indicating normal atrioventricular conduction.  Looking at the QRS complexes, there appears to be a normal axis with no significant deviation. The complexes are narrow, suggesting that the ventricles are being depolarized normally through the His-Purkinje system.  The ST segments do not show any significant elevation or depression in the leads provided, and the T waves appear normal. There are no signs of significant hypertrophy or acute ischemic changes on this portion of the ECG.  Based on this segment of the ECG, the preliminary diagnosis would be normal sinus rhythm without acute ischemic changes. However, it is important to consider the clinical context when interpreting an ECG, and to review all 12 leads for a complete analysis. Additionally, comparison with previous ECGs, if available, can provide information on whether there are any new changes or ongoing chronic conditions. | In this scenario, the patient presented with a loss of consciousness following a recent vaccination and had an episode of ventricular tachycardia (VT) transitioning to pulseless electrical activity (PEA), which required defibrillation. Given that resuscitation efforts were successful and the patient's heart rhythm has returned to what appears to be a normal sinus rhythm, as described from the ECG, the management would focus on post-cardiac arrest care and the possible causes of the event. Here's a structured approach:  1. Post-Resuscitation Care: This involves stabilization of the airway, breathing, and circulation. Ensure that the patient has a secure airway (intubate if not done already), is ventilated with appropriate oxygen to maintain normoxia, and has adequate perfusion (monitor blood pressure and urine output). Initiate therapeutic hypothermia if indicated.  2. Advanced Critical Care Monitoring: The patient should be admitted to the intensive care unit (ICU) for continuous monitoring of vital signs, cardiac telemetry, and frequent neurological checks due to the loss of consciousness and the need for resuscitation.  3. Neurological Assessment: Since the patient has not regained consciousness, a neurological consultation and imaging (such as a CT scan or MRI of the brain) should be performed to assess for any hypoxic-ischemic brain injury or other neurological causes for the loss of consciousness.  4. Etiology Workup: In parallel, it's important to identify the cause of the VT leading to PEA.  - Cardiac Causes: This could include ischemic heart disease, cardiomyopathies, or channelopathies. Cardiac biomarkers (troponins), echocardiography, and possibly a coronary angiogram would help evaluate these conditions.  - Non-Cardiac Causes: Electrolyte imbalances, drug effects, or other metabolic disturbances can lead to VT. A full metabolic panel, including magnesium and calcium levels, should be checked.  - Infectious Workup: Given the recent vaccine administration and fever, it would be prudent to consider myocarditis, which has been reported as a rare side effect of mRNA COVID-19 vaccines. Cardiac MRI can be diagnostic in such cases.  - Immunological Reaction: As the patient had a history of atopic dermatitis, there might be a predisposition to immune-mediated responses, although this is speculative. Consultation with an immunologist may be helpful.  5. Pharmacologic Therapy: Depending on the etiology, specific medications may be initiated. If myocarditis is suspected, the treatment is mainly supportive, but if an ischemic event is identified, antiplatelet and anticoagulant therapies may be warranted.  6. Vaccine-Related Reporting: This serious adverse event should be reported to the appropriate vaccine safety monitoring system in your country (such as VAERS in the United States), as it is critical for ongoing safety surveillance of vaccines.  7. Family Communication: Keep the family informed about the patient's status, the care being provided, and the ongoing investigations and treatment decisions.  8. Review and Consideration of DNR/DNI Status: Discuss the patient’s prognosis and potential outcomes with family and possibly consider advanced directives and code status in the context of the patient's clinical condition and the family's wishes.  Each step of this management should be taken with the involvement of a multidisciplinary team, including cardiologists, intensivists, neurologists, and possibly immunologists, along with nursing and allied health professionals to provide comprehensive care. |
| 2 | 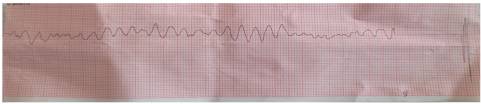  Emergency Medical Services (EMS) received a call from a bystander reporting a 46-year-old male who lost consciousness and was not breathing. The EMS team arrived at the scene within 4 minutes. The patient was found lying on the bed, and two bystanders had initiated CPR. The patient was unconscious, with no breathing and no pulse, and had mydriatic unresponsive pupils. The initial rhythm monitored on the cardiac defibrillator was above. | The image provided shows an ECG strip that is indicative of a tachyarrhythmia due to the fast heart rate. The QRS complexes appear wide and irregular, suggesting a ventricular rhythm. The lack of discernible P waves prior to each QRS complex, along with the broad and bizarre QRS morphology, are characteristic of ventricular tachycardia (VT).  VT is a type of cardiac arrhythmia that arises from improper electrical activity in the ventricles and can be life-threatening if not treated promptly. It can lead to hemodynamic instability and can degenerate into more dangerous rhythms such as ventricular fibrillation, which is a cardiac arrest rhythm, or can result in pulseless electrical activity (PEA) as apparently happened with this patient.  In an emergency setting, the immediate concern with VT, particularly if the patient is unstable, pulseless, or has collapsed, is to deliver advanced cardiac life support (ACLS) with immediate defibrillation and starting cardiopulmonary resuscitation (CPR) if there's no pulse. The management approach previously described would follow after the patient's heart rhythm has been restored and stabilized. | In this scenario, the patient is in cardiac arrest with a witnessed collapse and bystander CPR in progress. The described ECG findings in the previous image indicate ventricular tachycardia (VT), which is a shockable rhythm. Here’s how the EMS team should manage the patient according to Advanced Cardiac Life Support (ACLS) protocols:  1. Immediate Assessment: Verify the patient is unresponsive, not breathing, or only gasping and has no pulse. Confirm cardiac arrest.  2. Continue CPR: High-quality CPR should be continued with minimal interruptions. The current guidelines recommend a compression rate of 100 to 120 per minute and a depth of at least 2 inches (5 cm), allowing full chest recoil between compressions.  3. Defibrillation: As VT is a shockable rhythm, the patient should receive one shock. The defibrillator should be charged to the recommended energy setting (usually 120-200 Joules for biphasic defibrillators), and a defibrillation shock should be administered.  4. Resume CPR: Immediately after the shock, resume CPR beginning with chest compressions. Continue for 2 minutes before re-assessing the rhythm. Do not check for a pulse immediately after the shock; it takes time for the heart to re-establish a rhythm that can produce a pulse.  5. Airway Management: Secure the airway using the most appropriate device based on the provider's skill level. Provide ventilations at a rate of 10 breaths per minute if advanced airway (endotracheal tube or supraglottic device) is in place.  6. IV/IO Access: Establish intravenous (IV) or intraosseous (IO) access if not already in place.  7. Drug Therapy: Administer epinephrine 1 mg IV/IO as soon as possible and then every 3-5 minutes during the resuscitation. If the rhythm remains shockable at the next rhythm check, consider administering antiarrhythmic drugs such as amiodarone or lidocaine.  8. Rhythm and Pulse Check: After 2 minutes of CPR, check the rhythm. If still in VT or ventricular fibrillation (VF), deliver another shock. If an organized rhythm is present, check for a pulse.  9. Post-Resuscitation Care: If return of spontaneous circulation (ROSC) is achieved, begin post-resuscitation care, including maintaining oxygen saturation of 94-98%, considering advanced hemodynamic monitoring, and starting targeted temperature management if indicated.  10. Transport: Arrange for immediate transport to the nearest appropriate hospital while continuing to provide care en route.  11. Ongoing Assessment: Continue to monitor vital signs and ECG, secure airway and breathing, and administer medications as needed.  12. Documentation and Handover: Record all interventions and timings. Provide a detailed handover to the emergency department staff upon arrival.  Throughout this process, it’s crucial to minimize interruptions in chest compressions and to follow the current ACLS guidelines. This approach gives the patient the best chance of survival with good neurological outcomes. |
| 3 | 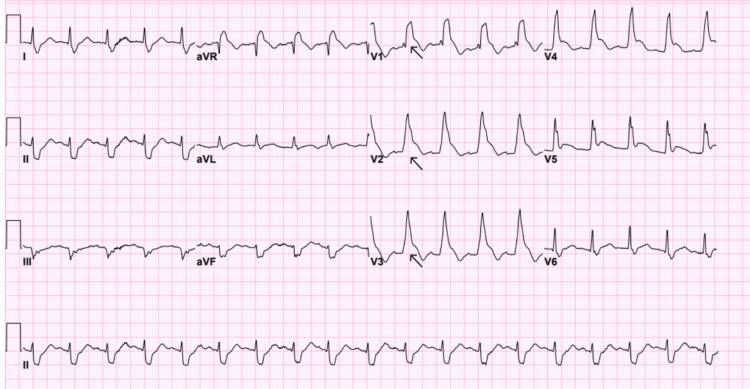  A 28-year-old male had a long-standing history of heavy smoking (two packs per day for 14 years) and recreational amphetamine use. He also had uncontrolled bronchial asthma that caused him to have shortness of breath with minimal exertion. On the day of admission, he developed severe chest pain, dyspnea, and vomiting while performing his morning exercise at 6:20 a.m. He was brought to the emergency department by his friend who witnessed the episode. Upon arrival, he was alert and oriented to time and place, with no active complaints. Physical examination findings were normal. Further history revealed recent amphetamine use, and a urine toxicology panel was positive for amphetamine.  An electrocardiography (ECG) performed on admission showed sinus tachycardia. However, after seven minutes of observation, he suddenly collapsed and became unresponsive. It was found that the patient had cardiac arrest, and the cardiac monitor showed ventricular fibrillation (VF). Advanced cardiovascular life support (ACLS) protocol was initiated, and he was immediately resuscitated with three cycles of CPR. An automated external defibrillator (AED) delivered three shocks of 200 J, 200 J, and 250 J, as well as a single dose of amiodarone and epinephrine, which resulted in the return of spontaneous circulation. The ECG taken upon his return was above. | This ECG demonstrates:  - Regular rhythm with a fast rate.  - No obvious P waves preceding the QRS complexes.  - The QRS complexes appear narrow.  - Tachycardia is present, but without a clear atrial activity preceding the QRS complexes.  Based on these findings, this ECG could represent supraventricular tachycardia (SVT). It is characterized by its abrupt onset, a heart rate that is typically between 150 and 250 beats per minute, and a narrow QRS complex, which suggests that the electrical impulse is still traveling normally through the ventricles.  To manage this, the underlying cause must be identified, and treatment should be aimed at controlling the rate, converting the rhythm to normal sinus rhythm, and preventing recurrence. Acute management may include vagal maneuvers, adenosine administration, or, in some cases, synchronized cardioversion if the patient is unstable.  However, further information about the patient's clinical status, history, and the context of this ECG reading are crucial for accurate diagnosis and management. | This patient presents a high-risk profile for cardiovascular complications given his history of heavy smoking, recreational amphetamine use, and uncontrolled bronchial asthma. The initial presentation of chest pain, dyspnea, and vomiting with a subsequent collapse and cardiac arrest due to ventricular fibrillation (VF) suggest an acute coronary syndrome (ACS), possibly exacerbated by amphetamine-induced coronary vasospasm.  The patient's return of spontaneous circulation (ROSC) after ACLS is positive, but the subsequent ECG is suggestive of a supraventricular tachycardia (SVT), which could be secondary to ischemia, electrolyte imbalance, or the direct effect of amphetamines.  Management should now include:  1. Post-Cardiac Arrest Care: The patient should be stabilized with continuous monitoring of vital signs and cardiac rhythm. Post-ROSC care includes optimizing ventilation and oxygenation, maintaining a systolic BP >90 mmHg, and considering targeted temperature management.  2. Further Assessment: This includes a complete blood count, comprehensive metabolic panel, cardiac enzymes, and coagulation profile. Assess for signs of heart failure or cardiogenic shock.  3. Cardiology Consultation: Urgent consultation with a cardiologist is advised for further evaluation and management, which may include coronary angiography to evaluate for occlusive coronary artery disease.  4. Imaging: A bedside echocardiogram to assess cardiac function and look for wall motion abnormalities indicative of ischemia should be performed.  5. Drug and Toxin Management: Given the patient’s amphetamine use, consider using benzodiazepines for sedation and to control any potential ongoing sympathetic overdrive.  6. Risk Factor Modification: Smoking cessation advice and substance abuse counseling should be part of the patient’s management plan, once stabilized.  7. Treatment of Bronchial Asthma: Reassessment and optimization of his asthma management are necessary to prevent future exacerbations.  8. Admission to ICU: The patient needs to be admitted to the intensive care unit (ICU) for further monitoring and management, given the high risk of recurrence of arrhythmias.  9. Secondary Prevention: Once the acute issue is addressed and the patient is stable, a long-term plan for secondary prevention of cardiovascular events should be discussed, which includes lifestyle modification, potential initiation of antiplatelet therapy, statins, and other medications as indicated by the underlying etiology and cardiology recommendations.  10. Follow-up and Rehabilitation: Plan for follow-up, including cardiology, primary care, and possibly a smoking cessation and substance abuse program. Consider referral to cardiac rehabilitation.  It’s critical to address all reversible causes and ensure comprehensive care given the complexity of this case. |
| 4 | 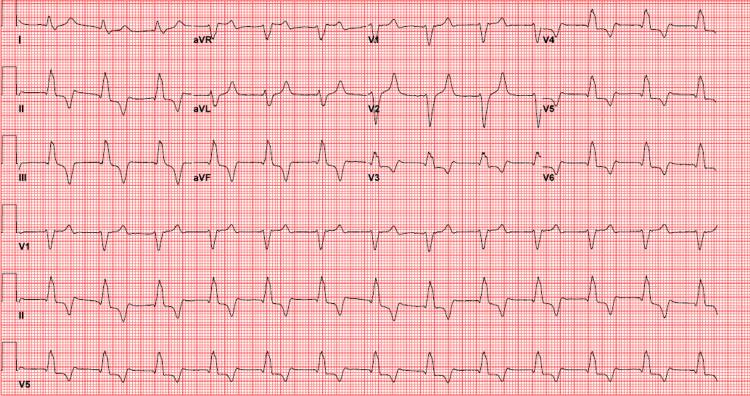  A 73-year-old female with a history of coronary artery disease, hypertension, and diabetes presented to the emergency department in cardiac arrest. According to Emergency Medical Services (EMS), she became unresponsive during breakfast at home with her son. Cardiopulmonary resuscitation (CPR) was initiated by her son, and upon arrival, EMS found the patient in pulseless electrical activity (PEA). The patient received 24 minutes of CPR and three doses of epinephrine, resulting in the return of spontaneous circulation (ROSC). A post-ROSC electrocardiogram (ECG) was obtained in the emergency department. | This ECG demonstrates:  - Sinus rhythm with P waves present before each QRS complex.  - A normal heart rate (the exact rate cannot be determined without the paper speed, but it appears to be within normal limits).  - Normal axis as evidenced by the QRS complex being positive in leads I and aVF.  - The QRS complexes appear narrow, indicating normal ventricular depolarization.  - There is no obvious ST-segment elevation or depression, T wave inversion, or significant Q waves to suggest ischemia or infarction on this tracing.  - There is no obvious sign of left or right ventricular hypertrophy.  - There is no sign of bundle branch block as the QRS duration appears normal.  Based on this ECG alone, the patient appears to have a normal sinus rhythm without acute ischemic changes. However, ECG interpretation should always be performed in the context of the patient's clinical presentation. Given this patient's history of a cardiac arrest with ventricular fibrillation, it is critical to perform a thorough evaluation for ischemic heart disease, including cardiac enzymes and potentially an echocardiogram or coronary angiography, as well as consider other causes such as cardiomyopathies, electrolyte disturbances, or drug effects. | In a post-cardiac arrest patient with a history of coronary artery disease, hypertension, and diabetes who presents with ROSC and a normal sinus rhythm on ECG, the management should be comprehensive and systematic:  1. Post-Cardiac Arrest Care: Initiate post-ROSC care, which includes optimizing hemodynamics, ventilation, and oxygenation. Target oxygen saturation should be between 94-98% and avoid hyperoxia. Ensure blood pressure is adequate, with a target systolic BP >90 mmHg or mean arterial pressure (MAP) >65 mmHg.  2. Secure Airway and Ventilation: If not already intubated, secure the airway with endotracheal intubation. Mechanical ventilation should be adjusted to achieve normocapnia unless specific contraindications are present.  3. Temperature Management: Initiate targeted temperature management (TTM) to maintain a core temperature between 32°C and 36°C for at least 24 hours.  4. Full Laboratory Workup: Obtain a complete set of labs, including cardiac biomarkers, complete blood count, comprehensive metabolic panel, coagulation profile, and blood gases. Monitor for electrolyte disturbances, particularly potassium and magnesium, given her cardiac history.  5. Cardiology Consultation and Further Cardiac Workup: Immediate cardiology consultation for potential coronary angiography to evaluate for acute coronary syndrome as a cause of her cardiac arrest. An echocardiogram should also be performed to assess left ventricular function and to look for wall motion abnormalities.  6. Glycemic Control: As the patient has diabetes, blood glucose levels should be closely monitored and controlled within a range that minimizes the risk of both hypoglycemia and significant hyperglycemia.  7. Neurological Assessment and Protection: Continuous monitoring of neurological status. Consideration for the use of sedation or even medically induced coma to protect the brain post-ROSC.  8. Consideration of Prognostication: It's often advised to delay neurological prognostication for at least 72 hours post-ROSC, particularly if TTM is utilized.  9. Determine the Cause of Arrest: Further investigation into the cause of the PEA arrest, such as a pulmonary embolism, myocardial infarction, or aortic dissection, should be considered, guided by clinical judgment, and additional findings.  10. Medication Review: Evaluate her medication regimen for adherence, potential drug interactions, and any need for adjustments given her medical history.  11. ICU Admission: Admit the patient to the intensive care unit for close monitoring, ongoing treatment, and frequent reassessment.  12. Family Communication and Support: Provide updates and support to her son and any other family members, explaining the interventions and the importance of each step.  The goal is to provide multidisciplinary care to address all potential reversible causes of the cardiac arrest, minimize the risk of recurrence, and maximize the patient's chance of neurological recovery. |
| 5 | 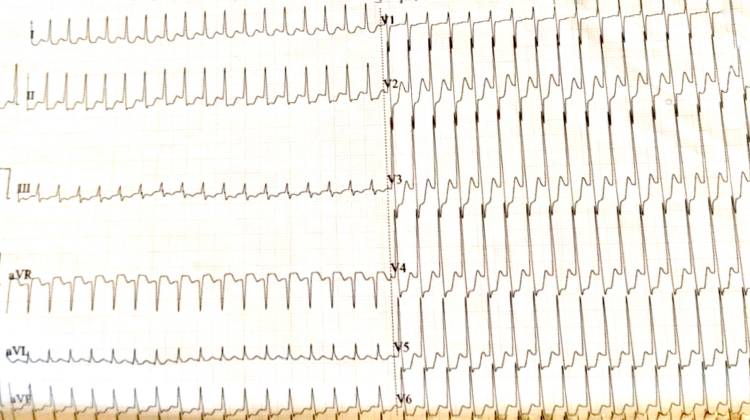  A 31-year-old female presented to the emergency department in a state of collapse with chief complaints of left-sided sharp chest pain and dyspnea lasting for two hours. The patient reported a mild dry cough since contracting COVID-19 five weeks prior, with a CT severity score of 5/25. All her investigations were within normal limits, and she was home quarantined as a result. There was no history of fever, loss of taste, or smell. The patient had no history of diabetes mellitus, hypertension, thyroid disorder, or any other comorbidities. She was a nonsmoker and had no history of supraventricular tachycardia or any other arrhythmias. The patient was immediately intubated for airway protection. | The ECG presented appears to show a wide complex tachycardia, which is most consistent with ventricular tachycardia (VT). Here are the key points to note:  1. Rate: The rate is very fast; however, the exact rate isn't measurable without the paper speed.  2. Rhythm: It looks to be a regular rhythm with consistent timing between the complexes.  3. QRS Complex: The complexes are wide, and the morphology is abnormal with no obvious preceding P wave, which is typical for VT.  4. P Wave: No discernible P waves can be seen before the QRS complexes, suggesting that the atria and ventricles are not functioning in a synchronized manner.  The diagnosis of VT is based on the presence of three or more consecutive premature ventricular complexes (PVCs) at a rate exceeding 100 beats per minute. This condition can be life-threatening, particularly if sustained, and requires immediate medical intervention.  In a clinical context, if the patient is hemodynamically unstable, immediate synchronized cardioversion is indicated. If the patient is stable, management may involve antiarrhythmic medication, such as amiodarone or lidocaine, and further evaluation of the underlying cause.  Since the scenario suggests that this ECG was obtained after ROSC, it's essential to continue post-resuscitation care and further work to identify the cause of the arrest, such as acute coronary syndrome, electrolyte imbalances, or other contributing factors. | The patient's presentation with sharp left-sided chest pain and dyspnea could be suggestive of a number of conditions, including pulmonary embolism (PE), pneumothorax, or pericarditis, among others. Given the recent COVID-19 infection, PE would be particularly concerning as COVID-19 can increase the risk of thromboembolic disease.  Based on the ECG findings consistent with ventricular tachycardia (VT), which was previously described, and the clinical picture, the following management steps should be considered:  1. Secure the Airway: Since the patient has already been intubated, ensure that the airway is managed properly with correct tube placement and adequate ventilation.  2. Oxygenation: Provide 100% oxygen initially and titrate to maintain an oxygen saturation of 94-98%.  3. Hemodynamic Stability: Assess the patient's blood pressure and heart rate. Since the ECG indicated VT, assess whether the patient is stable or unstable. If unstable, immediate synchronized cardioversion is indicated.  4. IV Access: If not already established, obtain intravenous access for administration of medications and fluids.  5. Rhythm Control: If the patient is in sustained VT but is hemodynamically stable, consider antiarrhythmic drugs such as amiodarone.  6. Laboratory Tests: Obtain a complete blood count, coagulation profile, basic metabolic panel, cardiac enzymes, D-dimer, and arterial blood gas analysis.  7. Imaging: Urgent imaging studies should be considered to rule out pulmonary embolism, pneumothorax, and other conditions. A CT pulmonary angiogram would be indicated to assess for PE.  8. Anticoagulation: If PE is suspected, and there are no contraindications, initiate anticoagulation therapy according to guidelines.  9. Specialty Consultation: Consult cardiology for the management of VT and pulmonology for the respiratory aspects and potential PE.  10. Monitoring: Continuous cardiac monitoring is essential to watch for recurrent arrhythmias.  11. Further Treatment: Address the cause of the VT once identified. If it is due to a PE, management will include anticoagulation and possibly thrombolysis or embolectomy if massive PE is confirmed.  12. Family Communication: Keep the family informed about the patient's condition, treatment plans, and progress.  13. Documentation: Ensure thorough documentation of the presentation, management steps taken, and the patient’s response to treatment.  The management of this patient should be multidisciplinary, involving emergency physicians, cardiologists, pulmonologists, and possibly intensivists for the comprehensive care required in such a critical presentation. |
| 6 | 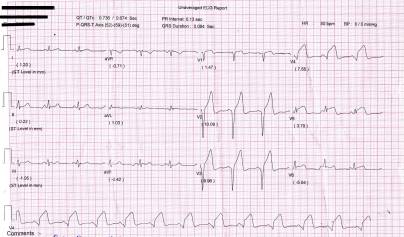  A 27-year-old male patient presented to the ED with complaints of retrosternal chest discomfort, difficulty in breathing, and profuse sweating for 6 hours prior to ED presentation. The patient also reported restlessness and three episodes of vomiting. On primary assessment, the patient was conscious with a patent airway. He had a respiratory rate of 30 breaths/min, maintaining an oxygen saturation of 85% on room air with bilateral crackles on auscultation. He was immediately transferred to a monitored bed and started on supplemental oxygen by face mask at 10 L/min, targeting a saturation of more than 95%. During the assessment of circulation, his pulse rate was 70 beats/min and blood pressure was 80/50 mmHg. Bedside ultrasonography revealed an ejection fraction of around 40%, with hypokinesia of the interventricular septum, left ventricular apex, and anterior wall. The inferior vena cava was 2.5 cm in diameter, with minimal respiratory variation and bilateral multiple B lines in all lung zones. Norepinephrine (at the rate of 10 mcg/min) was initiated. An ECG obtained after initial patient stabilization showed the ECG above. | This ECG shows:  - Rate: The heart rate appears to be regular on cursory inspection. To calculate it precisely, we would measure the number of large squares between two R waves and divide 300 by this number.  - Rhythm: There appears to be a P wave before each QRS complex, which suggests sinus rhythm, but measuring the PR interval would confirm this.  - Axis: The axis can be determined by looking at the QRS complexes in leads I and AVF to see if they are positive or negative.  - Hypertrophy: There are no overt signs of hypertrophy, but measuring the voltage in the limb and chest leads would be required to rule this out.  - ST Segment/T waves: There are noticeable abnormalities in the ST segments and T waves, particularly in leads V1 to V4, which may indicate ischemia or infarction.  - Q waves: There do not appear to be significant Q waves indicative of past infarctions.  - PR Interval: It seems to be consistent, but the exact duration cannot be determined without measuring.  - QT Interval: This should be measured to assess for abnormalities.  Preliminary Diagnosis: Without being able to perform precise measurements, this ECG suggests the possibility of an acute anterior wall myocardial infarction, given the ST elevations primarily in the precordial leads V1 to V4. However, a detailed analysis including rate, rhythm, axis, hypertrophy, and intervals is required to provide a more definitive interpretation. Additionally, clinical correlation and serial ECGs would be essential for an accurate diagnosis. If this ECG is from a patient presenting with chest pain or other related symptoms, this would be considered a medical emergency, and immediate management in the form of reperfusion therapy and supportive care would be indicated. | The patient is presenting with symptoms and signs suggestive of acute cardiopulmonary distress. Based on the history, examination, and investigations, the most likely diagnosis is an acute anterior myocardial infarction (MI) with resultant cardiogenic shock, as evidenced by hypotension, tachypnea, hypoxemia, and poor end-organ perfusion (as demonstrated by the sweating and vomiting).  Immediate Management should include:  1. Airway: Continue to monitor the airway to ensure it remains patent.  2. Breathing:  - Continue supplemental oxygen to maintain SpO2 >95%.  - Prepare for potential intubation if the patient's respiratory effort worsens or if hypoxemia is not corrected with non-invasive measures.  3. Circulation:  - Maintain the infusion of norepinephrine to support blood pressure.  - Consider fluid challenge vs. cautious diuresis based on the assessment of the patient's volume status. The IVC findings suggest euvolemia or possible hypervolemia, so diuresis may be warranted if the patient is not responsive to vasopressors.  - An intra-aortic balloon pump (IABP) or other mechanical circulatory support may be considered if blood pressure remains low despite medical therapy, indicating ongoing cardiogenic shock.  4. Drug Therapy:  - Aspirin, unless contraindicated, should be given immediately.  - Anticoagulation with heparin should be initiated to prevent further thrombus formation.  - Due to the ejection fraction being 40%, the use of beta-blockers should be cautious and might be contraindicated at this point because of the risk of worsening the cardiogenic shock.  5. Reperfusion Therapy:  - Urgent cardiology consultation for primary percutaneous coronary intervention (PCI), which is the preferred reperfusion strategy in acute MI if it can be performed in a timely manner.  - If PCI is not available within the recommended timeframe, consider thrombolytic therapy if there are no contraindications.  6. Monitoring and Support:  - Continuous monitoring of cardiac rhythm, respiratory rate, blood pressure, and oxygen saturation.  - Insertion of a urinary catheter to monitor urine output as a marker of end-organ perfusion.  - Serial cardiac enzymes to monitor the progression of the MI.  - Additional bedside ultrasonography to assess response to treatments and monitor for complications like ventricular septal rupture or free wall rupture.  7. Secondary Prevention and Risk Modification:  - After stabilization, medications such as ACE inhibitors, statins, and beta-blockers (when hemodynamically stable) should be initiated for long-term management.  8. Further Evaluation:  - Echocardiography for further assessment of cardiac function and structure.  - Coronary angiography as part of the PCI procedure or for diagnostic purposes if PCI is deferred.  - Laboratory tests including complete blood count, comprehensive metabolic panel, coagulation profile, and cardiac markers.  The patient should be managed with a multidisciplinary team approach, including emergency physicians, cardiologists, and possibly cardiac surgeons, depending on the evolution of the clinical picture and the response to initial management. |
| 7 | 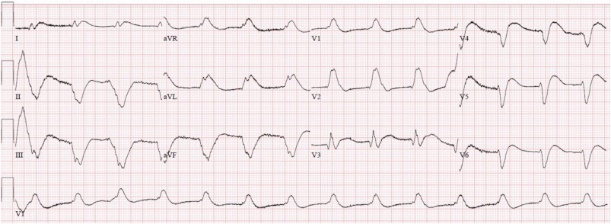  A 72-year-old man presented to the emergency department (ED) following a witnessed cardiac arrest. The initial rhythm was ventricular fibrillation (VF). After 35 minutes of chest compressions, four attempts at defibrillation, intubation, and multiple doses of epinephrine, return of spontaneous circulation (ROSC) was achieved in the ED. At this time, an electrocardiogram (ECG) was obtained. | This ECG shows:  - Rate: The heart rate appears fast; however, exact rate calculation requires counting the number of small squares between consecutive QRS complexes and dividing it into 1500, or the number of large squares into 300.  - Rhythm: There is no clear P wave before each QRS complex, suggesting an abnormal rhythm.  - Axis: The axis determination would require an assessment of the net QRS deflections in leads I and aVF.  - Hypertrophy: No signs of ventricular hypertrophy are evident without precise measurements.  - ST Segment/T waves: There are significant ST-segment elevations in leads V1-V4 and reciprocal changes (ST depression) in leads II, III, and aVF.  - Q waves: There are no obvious pathologic Q waves.  - PR Interval: Cannot be assessed properly without identifiable P waves.  - QT Interval: Cannot be assessed accurately without a clear baseline and T wave end.  Preliminary Diagnosis: The ECG suggests a Ventricular Tachycardia (VT) due to the wide QRS complexes at a fast rate with no clear P waves visible. The ST elevations in the precordial leads indicate that this is likely ischemic in nature. Given the clinical context, this could be an ischemic VT occurring in the setting of an acute anterior myocardial infarction. Immediate management should focus on stabilizing the rhythm, possibly using advanced cardiac life support (ACLS) protocols, including immediate cardioversion if the patient is hemodynamically unstable. Concurrently, addressing the underlying ischemia with urgent reperfusion therapy is critical. | Post-cardiac arrest care is critical for improving survival and neurological outcomes. This patient's ECG showing ventricular tachycardia in the setting of a recent cardiac arrest is concerning for ongoing myocardial ischemia. Here's how to proceed:  1. Post-ROSC Care:  - Ensure the patient remains hemodynamically stable; continue monitoring vital signs closely.  - Optimize ventilation and oxygenation post-intubation; aim for normocapnia and an oxygen saturation of 94-98%.  - Maintain or initiate sedation as appropriate.  - Start targeted temperature management (TTM) to maintain a core body temperature between 32°C and 36°C for at least 24 hours.  2. Cardiovascular Support:  - Assess for recurrent arrhythmias; prepare for immediate cardioversion if ventricular tachycardia recurs.  - Initiate or continue post-ROSC medications, like epinephrine infusion, to support blood pressure and heart rate as needed.  - Consider invasive hemodynamic monitoring to guide fluid and inotropic support.  - Assess for acute coronary syndrome (ACS) as a potential cause of the cardiac arrest.  - Urgent coronary angiography should be considered given the VF and the suggestive ischemic ECG changes.  - Re-evaluate medications, ensuring antiarrhythmics and other cardioprotective agents are administered as indicated.  3. Neurological Assessment:  - Perform a thorough neurological examination when feasible.  - Continue with sedation to prevent shivering and allow for TTM.  - Arrange for a neurological consult and consider neuroimaging if there are localizing signs or if the patient does not regain consciousness as expected after warming.  4. Investigations:  - Repeat ECGs to monitor for changes.  - Blood tests, including cardiac biomarkers, complete blood count, electrolytes, renal function, coagulation profile, and arterial blood gases.  - Echocardiography to evaluate cardiac function and look for structural abnormalities.  5. Further Management:  - Engage in a multi-disciplinary team discussion including cardiology, critical care, and possibly cardiothoracic surgery, depending on the findings and response to initial treatments.  - Discuss with family or surrogate decision-makers regarding the prognosis and ongoing care plans, including advanced directives and potential escalation or de-escalation of care.  6. Prevention of Complications:  - Gastric ulcer prophylaxis.  - Deep vein thrombosis prophylaxis.  - Regular turning and care to prevent pressure ulcers.  - Glycemic control within a target range to prevent hyperglycemia.  7. Documentation:  - Document the resuscitation timeline and interventions, including the total duration of CPR, medications given, and shocks delivered.  This case should be managed in an intensive care setting, with close monitoring and frequent reassessments, given the patient's age and the prolonged resuscitation period. |
| 8 | 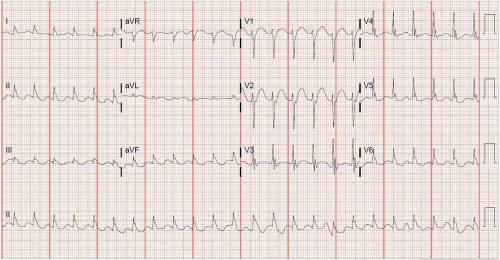  A previously healthy 33-year-old male presented to the emergency department after suffering from a cardiac arrest at home. According to his family, he had been in his normal state of health until one week prior when he reported fatigue. The patient was a bodybuilder and had a history of prolonged anabolic steroid use. He had recently started using bovine levothyroxine not intended for human consumption to enhance his performance. On the day of presentation, the patient began experiencing difficulty breathing, became unresponsive, and developed cardiopulmonary arrest followed immediately by bystander cardiopulmonary resuscitation (CPR). Upon arrival of emergency medical services (EMS), the patient was found to be in ventricular fibrillation (VF) and was defibrillated. CPR was continued and he received 1 mg of epinephrine, resulting in the return of spontaneous circulation (ROSC). A laryngeal mask airway was placed by EMS. On arrival to the emergency department, the patient remained unresponsive with an initial heart rate of 140, blood pressure of 90/60 mmHg, respirations via assisted ventilation at a rate of 12, and a temperature of 36.4°C. He was started on norepinephrine at a rate of 0.05 mcg/kg/min with improvement in blood pressure to 115/71. The initial ECG was above. | This ECG demonstrates:  - Rate: Fast heart rate; exact rate not calculable without the standard paper speed reference, but there are more than five QRS complexes in a 5-square segment, suggesting a rate over 100 beats per minute.  - Rhythm: The rhythm is irregularly irregular, which is suggestive of atrial fibrillation.  - P Waves: There are no discernible P waves, consistent with atrial fibrillation.  - QRS Complexes: QRS complexes appear narrow, which suggests that the ventricles are being depolarized normally through the His-Purkinje system.  - ST Segments: There are no significant ST elevations or depressions visible on this tracing.  - T Waves: The T waves appear normal in the leads shown.  - PR Interval: Cannot be measured due to the absence of P waves.  - QT Interval: QT interval appears normal, although exact measurement is not possible from the image.  The preliminary diagnosis from this ECG is atrial fibrillation with a rapid ventricular response. This condition can be associated with a variety of symptoms and may increase the risk for thromboembolic events. Management typically includes rate control with medications like beta-blockers or calcium channel blockers, rhythm control (potentially including cardioversion if the onset is known to be recent), and anticoagulation to reduce the risk of stroke. Given the patient's history of a recent cardiac arrest, the context of this ECG would influence immediate management decisions, including the need for further cardiology evaluation and potential intervention. | The patient has a history suggestive of anabolic steroid and thyroid hormone misuse, both of which can contribute to cardiac arrhythmias and cardiomyopathy. The ECG findings previously described as atrial fibrillation with a rapid ventricular response are also concerning, especially in the context of his history. Here's how to manage the patient:  1. Advanced Cardiac Life Support (ACLS): Continue to follow ACLS protocols. Given that the patient has achieved ROSC but is hemodynamically unstable, maintain vasopressor support to keep mean arterial pressure (MAP) above 65 mmHg.  2. Secure Airway: Replace the laryngeal mask airway with endotracheal intubation for a more secure airway and to provide controlled ventilation. Ensure proper endotracheal tube placement with confirmation by capnography and chest X-ray.  3. Breathing and Oxygenation: Titrate oxygen to maintain SpO2 between 94-98% to avoid hyperoxia. Set appropriate ventilator settings to maintain normocapnia and avoid lung injury.  4. Circulation: Continue norepinephrine to support blood pressure and consider an echocardiogram to evaluate cardiac function and structure in light of his steroid use and recent cardiovascular collapse.  5. Temperature Management: Continue to monitor temperature closely. If the patient remains unresponsive, consider targeted temperature management (TTM) to maintain a core temperature between 32°C and 36°C for 24 hours.  6. Electrolytes and Metabolic: Monitor and correct electrolytes, acid-base, and glucose abnormalities. Anabolic steroid and thyroid hormone abuse can lead to significant metabolic disturbances.  7. Toxicology: Consult toxicology for management of potential complications of anabolic steroid and thyroid hormone misuse. Bovine levothyroxine, especially, is not intended for human use and may have unpredictable effects.  8. Cardiology and Endocrinology Consults: Request consults for further management of the patient's tachyarrhythmia and for the endocrine disturbances from thyroid hormone misuse.  9. Laboratory Tests: Obtain a full lab panel including thyroid function tests, cardiac enzymes, complete blood count, coagulation profile, renal and liver function tests, and a toxicology screen.  10. Imaging: Perform a chest X-ray to check for proper line and tube placement and to assess for pulmonary edema or other pathologies.  11. Neurological Assessment: As the patient is unresponsive, conduct a neurological exam and consider imaging such as a head CT to rule out hypoxic-ischemic injury or other intracranial processes.  12. Family Communication: Update the family on the patient's status, the treatments being provided, and the importance of providing all relevant information about the patient's medical history and substance use.  13. Documentation: Keep meticulous documentation of the resuscitation effort, the patient's response, and the ongoing management plan.  14. Plan for Potential Complications: Be vigilant for complications associated with prolonged resuscitation, anabolic steroid use, and hyperthyroidism, including arrhythmias, myocardial infarction, thromboembolism, and multiorgan failure.  This patient requires intensive monitoring and management in an ICU setting, with multidisciplinary involvement for his complex clinical situation. |
| 9 | 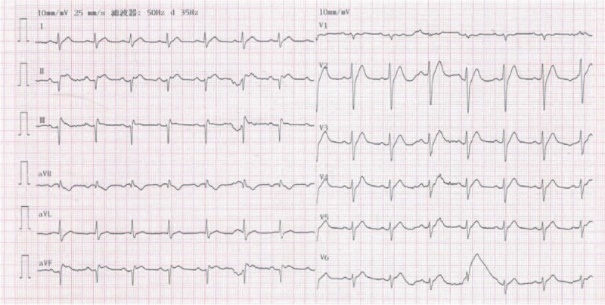  A 40-year-old man developed sudden precordial dull and unrelieved pain accompanied by sweating for 1 hour. When he arrived at the emergency department, he suddenly lost consciousness, his breathing halted, and he experienced cardiac arrest. The doctors urgently performed cardiopulmonary resuscitation (CPR) on him, including chest compressions, three times electric defibrillation, tracheal intubation, ventilator-assisted breathing, and dopamine intravenous infusion. After about 10 minutes, his respiration and heartbeat recovered, but he remained unconscious with double pupil dilation of 7 mm. His vital signs revealed a body temperature of 36.2°C, a heart rate of 89 beats per minute, a respiration rate of 32 breaths per minute, and a blood pressure of 95/52 mmHg. The patient had a history of hyperlipidemia for 1 year. The first ECG was above. | The ECG shows:  - Rate: The heart rate is fast; the QRS complexes are closely spaced.  - Rhythm: The rhythm appears to be irregularly irregular, which suggests atrial fibrillation.  - P Waves: P waves are not discernible, consistent with atrial fibrillation.  - QRS Complex: The QRS complexes appear narrow, which is typical for supraventricular rhythms, and they follow the irregular rhythm without a clear pattern, reinforcing the diagnosis of atrial fibrillation.  - ST Segments: No significant ST elevation is seen. There may be some ST depression, but it's not clearly pathological on this tracing.  - T Waves: T waves are present, but specifics on morphology are unclear without higher-quality tracing.  - PR Interval: Cannot be measured due to the absence of P waves.  - QT Interval: The QT interval appears to be within normal limits, but this is difficult to measure accurately without clear demarcation of the end of the T wave.  The most likely preliminary diagnosis is atrial fibrillation with a rapid ventricular response. Further clinical correlation and lab work are necessary to guide the treatment, which typically includes rate control, rhythm control, and anticoagulation to prevent thromboembolic events. Given the patient's history of cardiac arrest, it would be crucial to investigate the underlying causes of his atrial fibrillation and to assess for any associated structural heart disease. | This patient's presentation is highly suggestive of an acute myocardial infarction (AMI) leading to cardiac arrest. Post-resuscitation, the ECG findings previously described as atrial fibrillation with a rapid ventricular response further complicate his clinical picture. Here is an appropriate management plan:  1. Post-ROSC Care: Follow advanced post-cardiac arrest care which includes securing the airway, optimizing ventilation, and supporting circulation.  2. Airway and Breathing: Confirm endotracheal tube placement with capnography and a chest X-ray. Adjust ventilator settings to maintain normocapnia and appropriate oxygenation, avoiding hyperoxia.  3. Circulation: Continue dopamine or switch to another vasopressor or inotrope if necessary to maintain adequate blood pressure and perfusion. Obtain central venous access and an arterial line for close hemodynamic monitoring.  4. Coronary Reperfusion: Given the history and presentation suggestive of AMI, urgent cardiology consultation is necessary for potential coronary angiography and reperfusion therapy, which may include percutaneous coronary intervention (PCI).  5. Temperature Management: Initiate targeted temperature management to maintain a core temperature between 32-36°C, especially since the patient remains unconscious. This could help reduce neurological damage.  6. Neurological Care: Consult neurology for the evaluation of the patient's unconscious state and fixed, dilated pupils. Consider a head CT to rule out intracranial hemorrhage, especially before starting any anticoagulation.  7. Laboratory Workup: Obtain a full set of labs, including cardiac biomarkers, complete blood count, basic metabolic panel, coagulation profile, arterial blood gases, and lipid profile.  8. Electrolytes and Acid-Base Management: Monitor and correct any electrolyte imbalances or acid-base disorders, which can be critical in the post-arrest period.  9. Glycemic Control: Manage the patient's blood glucose levels within a normal range, avoiding both hyperglycemia and hypoglycemia.  10. Anticoagulation: Carefully consider anticoagulation for atrial fibrillation with a rapid ventricular response, balancing the risk of thromboembolic events with the potential risk of bleeding after recent CPR.  11. Echocardiogram: Perform an echocardiogram to assess the cardiac function, wall motion abnormalities, and any complications of AMI.  12. Family Communication: Update the family on the patient's condition, the care being provided, and discuss the potential prognosis.  13. Documentation: Document all interventions, clinical findings, and family discussions thoroughly.  14. Consideration for Support Services: If the patient has sustained significant neurological injury, discuss with the family the potential need for long-term care and the involvement of social services or palliative care if appropriate.  15. Continuous Monitoring: The patient should be in a critical care setting with continuous hemodynamic, respiratory, and neurological monitoring.  16. Review of Medications: Assess the patient's medication regimen, especially lipid-lowering therapy, and adjust as necessary in the context of acute care.  This patient requires intensive care and a multidisciplinary approach, involving emergency medicine, cardiology, neurology, and possibly other specialties as his condition evolves. |
| 10 | 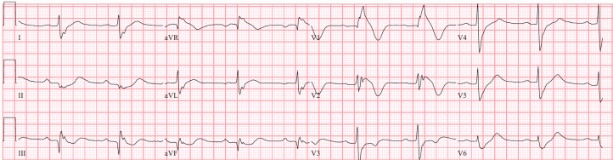  A 74-year-old man was transferred to a Level I trauma center emergency department (ED) following a low-speed motor vehicle collision from a small local community hospital. According to the report from emergency medical services (EMS) personnel, the patient was initially driving very slowly behind another vehicle. When the other vehicle stopped, the patient's vehicle collided with the stopped vehicle. Bystanders noted the patient to be slumped over and unresponsive in his vehicle, at which time EMS was called. Upon arrival, EMS found the patient to be unresponsive and pulseless, with their initial ECG demonstrating torsades de pointes. Cardiopulmonary resuscitation was initiated. Paramedics administered epinephrine, magnesium, and amiodarone. The patient experienced a return of spontaneous circulation and was transported to the hospital. The initial ECG was above. | The ECG presents with the following features:  - Rate: The heart rate is regular but bradycardic (less than 60 bpm).  - Rhythm: Normal sinus rhythm with consistent P waves before each QRS complex.  - Axis: The QRS complex axis is normal with positive deflections in leads I and aVF.  - Hypertrophy: There are no overt signs of hypertrophy.  - ST Segment/T waves: There is ST elevation in leads II, III, and aVF, which are indicative of an inferior wall myocardial infarction. There are reciprocal changes (ST depressions) in the lateral leads I and aVL.  - Q waves: Pathologic Q waves are not clearly visible on this tracing.  - PR Interval: Appears to be normal, but precise measurement is required.  - QT Interval: Cannot be accurately assessed without exact measurements.  Preliminary Diagnosis: The ECG suggests an acute inferior wall myocardial infarction, based on the ST elevations in the inferior leads (II, III, and aVF). Immediate clinical correlation and further management, including reperfusion therapy (preferably primary percutaneous coronary intervention, if timely), are indicated. The bradycardia could be a reflex response to the inferior MI or due to increased vagal tone, often seen in acute inferior infarctions. It is important to assess the patient for any hemodynamic instability and treat accordingly. | This patient has had a witnessed cardiac arrest in the context of a motor vehicle collision (MVC), with initial ECG showing torsades de pointes, a form of polymorphic ventricular tachycardia often associated with a prolonged QT interval. The current ECG suggests an acute inferior myocardial infarction (MI). The MVC may have been a result of the cardiac event rather than the cause of it. Management should include:  1. Post-Resuscitation Care:  - Admit to an intensive care unit for close hemodynamic and rhythm monitoring.  - Optimize ventilation and oxygenation; wean oxygen to keep SpO2 between 94-98%.  - Sedation as needed for comfort, especially if mechanical ventilation is ongoing.  - Continue post-ROSC care according to the latest ACLS guidelines.  - Initiate targeted temperature management (TTM) if indicated.  2. Cardiovascular Support:  - Continue monitoring for recurrent ventricular arrhythmias and be prepared for immediate intervention.  - Assess the need for ongoing vasopressor support and adjust based on blood pressure and signs of perfusion.  - Initiate anti-arrhythmic drugs as indicated, considering the history of torsades de pointes.  3. Reperfusion Therapy:  - Urgent cardiology consultation for consideration of coronary angiography and possible percutaneous coronary intervention (PCI), given the evidence of an inferior MI.  - Continue with aspirin and anticoagulation unless contraindicated.  4. Neurological Evaluation:  - Neurological status assessment once the patient is stabilized.  - Further imaging and diagnostic tests as indicated to rule out any traumatic brain injury from the MVC.  5. Trauma Evaluation:  - Full secondary survey to assess for any other injuries related to the MVC.  - Imaging studies, such as X-rays, CT scans as indicated based on the trauma assessment.  6. Laboratory Tests:  - Complete blood count, comprehensive metabolic panel, troponin levels, coagulation profile, arterial blood gas analysis, and toxicology screen.  7. Electrolyte Repletion:  - Monitor and correct electrolyte imbalances, especially magnesium and potassium, which are important for stabilizing the myocardium and preventing recurrent arrhythmias.  8. Consultation and Follow-up:  - Cardiology for the MI and arrhythmia.  - Trauma surgery or orthopedics if any injuries are found.  - Neurology if there is concern for a neurological deficit.  9. Patient and Family Communication:  - Provide updates to the family regarding the patient's status and involve them in care decisions as appropriate.  10. Documentation:  - Document the resuscitation timeline and interventions meticulously, including the total duration of CPR, medications given, shocks delivered, and post-ROSC care.  In parallel, consider the possibility of a secondary cause for the torsades de pointes, such as a congenital long QT syndrome, though it is less likely in this age group, or acquired causes such as electrolyte disturbances or medications. This patient's care will be complex and will require a multidisciplinary approach. |

*Cases taken from OSCE sample questions

** Cases obtained from Pubmed
